# Selective formation of [Au_23_(SPh^*t*^Bu)_17_]^0^, [Au_26_Pd(SPh^*t*^Bu)_20_]^0^ and [Au_24_Pt(SC_2_H_4_Ph)_7_(SPh^*t*^Bu)_11_]^0^ by controlling ligand-exchange reaction†

**DOI:** 10.1039/d2sc00423b

**Published:** 2022-03-30

**Authors:** Yuichi Negishi, Hikaru Horihata, Ayano Ebina, Sayuri Miyajima, Mana Nakamoto, Ayaka Ikeda, Tokuhisa Kawawaki, Sakiat Hossain

**Affiliations:** Department of Applied Chemistry, Faculty of Science, Tokyo University of Science Kagurazaka, Shinjuku–ku Tokyo 162-8601 Japan negishi@rs.tus.ac.jp; Research Institute for Science & Technology, Tokyo University of Science Kagurazaka, Shinjuku-ku Tokyo 162-8601 Japan

## Abstract

To use atomically precise metal nanoclusters (NCs) in various application fields, it is essential to establish size-selective synthesis methods for the metal NCs. Studies on thiolate (SR)-protected gold NCs (Au_*n*_(SR)_*m*_ NCs) revealed that the atomically precise Au_*n*_(SR)_*m*_ NC, which has a different chemical composition from the precursor, can be synthesized size-selectively by inducing transformation in the framework structure of the metal NCs by a ligand-exchange reaction. In this study, we selected the reaction of [Au_25_(SC_2_H_4_Ph)_18_]^−^ (SC_2_H_4_Ph = 2-phenylethanethiolate) with 4-*tert*-butylbenzenethiol (^*t*^BuPhSH) as a model ligand-exchange reaction and attempted to obtain new metal NCs by changing the amount of thiol, the central atom of the precursor NCs, or the reaction time from previous studies. The results demonstrated that [Au_23_(SPh^*t*^Bu)_17_]^0^, [Au_26_Pd(SPh^*t*^Bu)_20_]^0^ (Pd = palladium) and [Au_24_Pt(SC_2_H_4_Ph)_7_(SPh^*t*^Bu)_11_]^0^ (Pt = platinum) were successfully synthesized in a high proportion. To best of our knowledge, no report exists on the selective synthesis of these three metal NCs. The results of this study show that a larger variety of metal NCs could be synthesized size-selectively than at present if the ligand-exchange reaction is conducted while changing the reaction conditions and/or the central atoms of the precursor metal NCs from previous studies.

## Abstract 2

This study succeeded in obtaining three new thiolate protected metal nanoclusters by changing the ligand-exchange condition from previous studies.

## Graphical abstract

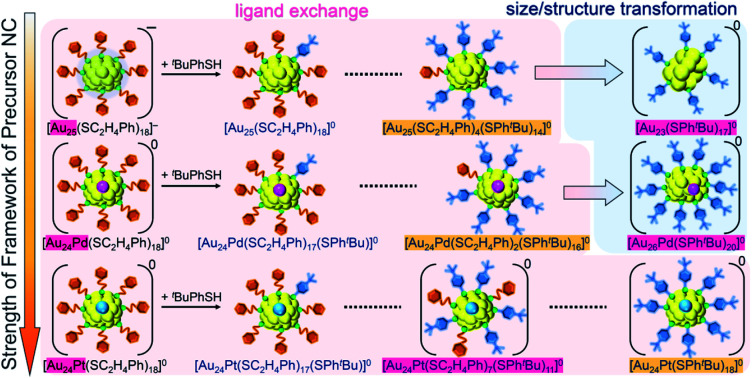

## Introduction

To use atomically precise metal nanoclusters (NCs) in various application fields^1–6^ while taking advantage of their size-specific physicochemical properties,^7–9^ it is essential to establish size-selective synthesis methods for the metal NCs. Studies on thiolate (SR)-protected gold NCs (Au_*n*_(SR)_*m*_ NCs),^10–18^ which are the most studied metal NCs, have revealed that it is extremely effective to expose the mixture of Au_*n*_(SR)_*m*_ NCs with a distribution in chemical composition to harsh conditions and thereby converge them to stable NCs to selectively obtain atomically precise Au_*n*_(SR)_*m*_ NC.^19,20^ Recent studies revealed that ligand exchange of the obtained atomically precise Au_*n*_(SR)_*m*_ NC with other ligands with a different bulkiness also yields atomically precise Au_*n*_(SR)_*m*_ NC with a different chemical composition.^21^ In the latter method, which is termed ligand-exchange-induced size/structure transformation (LEIST), new atomically precise Au_*n*_(SR)_*m*_ NCs and related NCs are size-selectively synthesized by inducing transformation in the framework structure of precursor NCs by ligand exchange.^22–24^

**Scheme 1:**
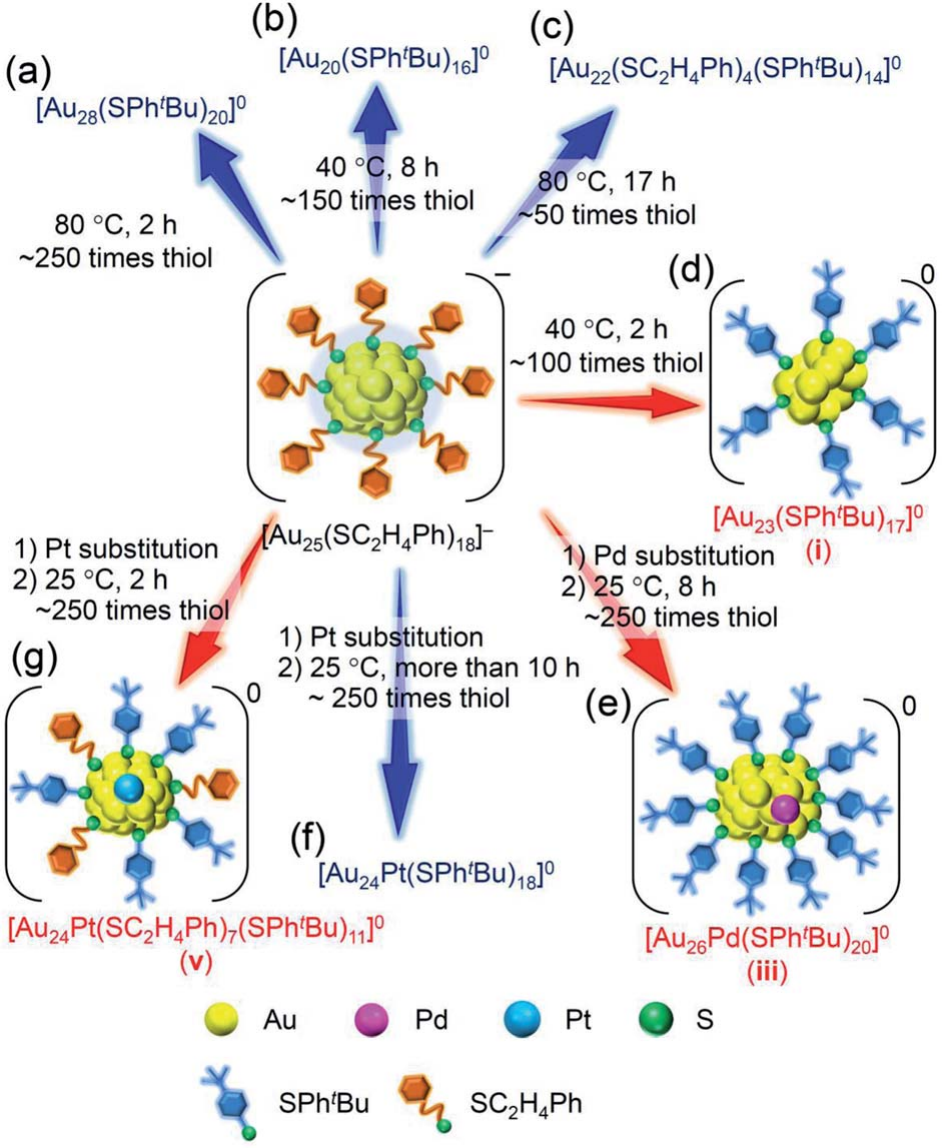
Experimental conditions for the formation of each metal NC: (a) [Au_28_(SPh^*t*^Bu)_20_]^0^,^25^ (b) [Au_20_(SPh^*t*^Bu)_16_]^0^,^26^ (c) [Au_22_(SC_2_H_4_Ph)_4_(SPh^*t*^Bu)_14_]^0^,^27^ (d) [Au_23_(SPh^*t*^Bu)_17_]^0^ (**i**; this work), (e) [Au_26_Pd(SPh^*t*^Bu)_20_]^0^ (**iii**; this work), (f) [Au_24_Pt(SPh^*t*^Bu)_18_]^0^,^35^ and (g) [Au_24_Pt(SC_2_H_4_Ph)_7_(SPh^*t*^Bu)_11_]^0^ (**v**; this work).

For such LEIST, if the reaction is carried out under different conditions from the reported condition, it may be possible to proceed with a reaction by a different pathway and/or obtain products with a different chemical composition. In the reaction between [Au_25_(SC_2_H_4_Ph)_18_]^−^ (SC_2_H_4_Ph = 2-phenylethanethiolate, Scheme S1(a) and S2(a)†) and 4-*tert*-butylbenzenethiol (^*t*^BuPhSH), [Au_28_(SPh^*t*^Bu)_20_]^0^ (SPh^*t*^Bu = 4-*tert*-butylbenzenethiolate, Scheme S1(b) and S2(b)†) is size-selectively synthesized at 80 °C (Scheme 1(a)),^25^ and [Au_20_(SPh^*t*^Bu)_16_]^0^ is also formed in ∼10% yield at 40 °C (Scheme 1(b) and S2(c)†).^26^ Even at 80 °C, when the amount of ^*t*^BuPhSH is reduced to ∼50 times the amount of SC_2_H_4_Ph in [Au_25_(SC_2_H_4_Ph)_18_]^−^ ([^*t*^BuPhSH]/[SC_2_H_4_Ph] ≅ 50), [Au_22_(SC_2_H_4_Ph)_4_(SPh^*t*^Bu)_14_]^0^ is produced at a high proportion (Scheme 1(c) and S2(d)†).^27^ Based on these facts, it is expected that further modification of the reaction conditions (*e.g.*, amount of thiol, temperature, reaction time) in LEIST will result in the size-selective synthesis of new Au_*n*_(SR)_*m*_ NCs, which has not been reported previously.

In addition to such changes in reaction conditions, heteroatom substitutions (Scheme S2(e) and (f)†)^28–31^ in the precursor Au_*n*_(SR)_*m*_ NCs may also induce changes in the reaction pathway and/or chemical composition of the products.^32,33^ Indeed, our previous studies have shown that when [Au_24_Pt(SC_2_H_4_Ph)_18_]^0^ (Pt = platinum; Scheme S2(f)†),^34^ in which the central Au atom of [Au_25_(SC_2_H_4_Ph)_18_]^−^ is replaced with a atom, is reacted with ^*t*^BuPhSH, the ligand exchange proceeds while keeping a metal core size, unlike for [Au_25_(SC_2_H_4_Ph)_18_]^−^, and thereby [Au_24_Pt(SPh^*t*^Bu)_18_]^0^, in which all ligands are exchanged with SPh^*t*^Bu, can be size-selectively synthesized.^35^ This change in reaction pathway because of Pt substitution is related mainly to the difference in the strength of the framework between Au_25_(SR)_18_ and Au_24_Pt(SR)_18_. Because [Au_24_Pt(SC_2_H_4_Ph)_18_]^0^ has a stronger framework than [Au_25_(SC_2_H_4_Ph)_18_]^−^,^36^ it can maintain its framework structure even when the ligand is exchanged from SC_2_H_4_Ph to SPh^*t*^Bu, and thereby [Au_24_Pt(SPh^*t*^Bu)_18_]^0^ was synthesized in the reaction between [Au_24_Pt(SC_2_H_4_Ph)_18_]^0^ and ^*t*^BuPhSH. Density functional theory (DFT) calculations suggest that Au_24_Pd(SR)_18_ (Pd = palladium; Scheme S2(e)†),^30^ in which the central Au atom of [Au_25_(SC_2_H_4_Ph)_18_]^−^ is replaced with a Pd atom, has a framework strength between Au_25_(SR)_18_ and Au_24_Pt(SR)_18_.^36^ Therefore, it is expected that when [Au_24_Pd(SC_2_H_4_Ph)_18_]^0^ is reacted with ^*t*^BuPhSH, the reaction proceeds differently from the case when [Au_25_(SC_2_H_4_Ph)_18_]^−^ or [Au_24_Pt(SC_2_H_4_Ph)_18_]^0^ is used as the precursor NC, and thereby new metal NCs can be synthesized size-selectively.

**Scheme 2:**
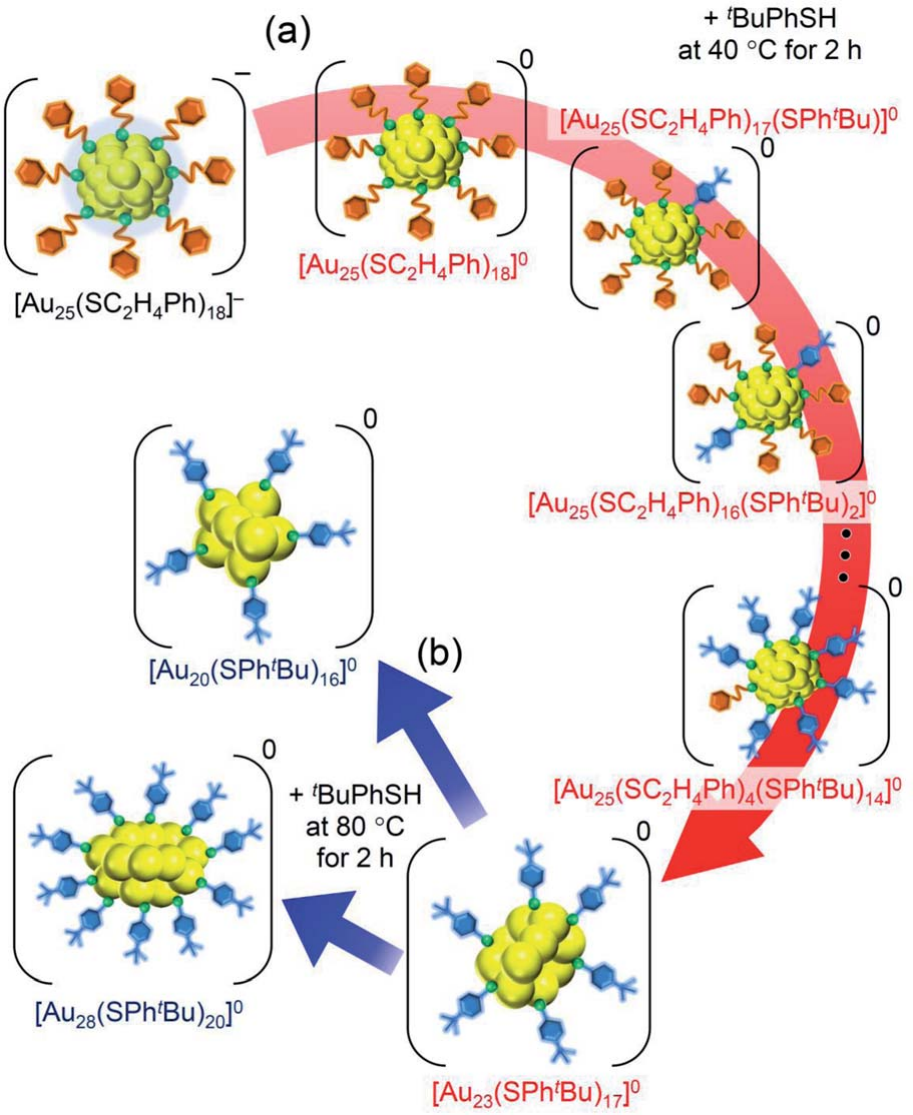
Schematic illustration of transformation from (a) [Au_25_(SC_2_H_4_Ph)_18_]^−^ to [Au_23_(SPh^*t*^Bu)_17_]^0^ (**i**) and (b) [Au_23_(SPh^*t*^Bu)_17_]^0^ (**i**) to [Au_20_(SPh^*t*^Bu)_16_]^0^ and [Au_28_(SPh^*t*^Bu)_20_]^0^.

**Scheme 3:**
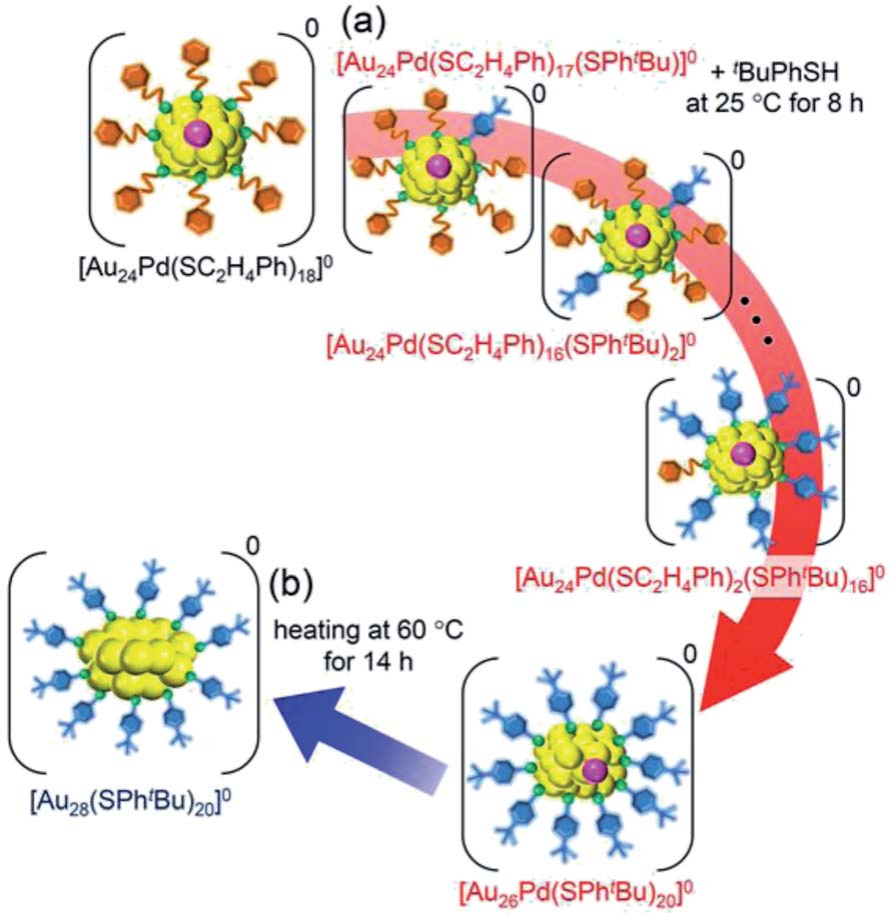
Schematic illustration of the transformation from (a) [Au_24_Pd(SC_2_H_4_Ph)_18_]^0^ to [Au_26_Pd(SPh^*t*^Bu)_20_]^0^ (**iii**) and (b) [Au_26_Pd(SPh^*t*^Bu)_20_]^0^ (**iii**) to [Au_28_(SPh^*t*^Bu)_20_]^0^.

The purpose of this study was to increase the variety of atomically precise metal NCs that can be synthesized size-selectively. Among the Au_*n*_(SR)_*m*_ NCs, [Au_25_(SC_2_H_4_Ph)_18_]^−^ is one of the most studied NCs,^37,38^ and ^*t*^BuPhSH has been used frequently as an exchanged ligand in previous LEIST experiments.^12,23,35^ Therefore, in this study, we selected the reaction of [Au_25_(SC_2_H_4_Ph)_18_]^−^ with ^*t*^BuPhSH as a model reaction and attempted to obtain new NCs by changing the reaction conditions or the central atom of the precursor NCs. Specifically, the following changes were applied in previous reports:^26,35^ (1) the amount of ^*t*^BuPhSH was reduced in the reaction at 40 °C (Scheme 1(d)), (2) the central Au atom of [Au_25_(SC_2_H_4_Ph)_18_]^−^ was substituted with a Pd atom ([Au_24_Pd(SC_2_H_4_Ph)_18_]^0^) (Scheme 1(e)), (3) the reaction time in the reaction between [Au_24_Pt(SC_2_H_4_Ph)_18_]^0^ and ^*t*^BuPhSH was shortened (Scheme 1(g)). These attempts led to the formation of [Au_23_(SPh^*t*^Bu)_17_]^0^ (Scheme 1(d)), [Au_26_Pd(SPh^*t*^Bu)_20_]^0^ (Scheme 1(e)) and [Au_24_Pt(SC_2_H_4_Ph)_7_(SPh^*t*^Bu)_11_]^0^ (Scheme 1(g)) in a high proportion. To the best of our knowledge, no report exists on the selective synthesis for these three metal NCs. These results demonstrate that a larger variety of atomically precise metal NCs can be synthesized size-selectively by ligand exchange than at present if the ligand-exchange reaction is conducted while changing the reaction conditions and/or central atoms of the precursor metal NCs from previous studies.

## Results and discussion

### Reaction between [Au_25_(SC_2_H_4_Ph)_18_]^−^ and ^*t*^BuPhSH

Previous studies on this reaction system have shown that [Au_28_(SPh^*t*^Bu)_20_]^0^ can be synthesized selectively when the reaction proceeds at 80 °C with a ratio of [^*t*^BuPhSH]/[SC_2_H_4_Ph] ≅ 250,^25^ whereas [Au_20_(SPh^*t*^Bu)_16_]^0^ can be formed in ∼10% yield when the reaction proceeds at 40 °C with a ratio of [^*t*^BuPhSH]/[SC_2_H_4_Ph] ≅ 150.^26^ In this study, we conducted the reaction at 40 °C with a ratio of [^*t*^BuPhSH]/[SC_2_H_4_Ph] ≅ 100 (Scheme 1(d)) to allow the reaction to proceed more slowly than in a previous report and thereby create a novel metal NC.

**Fig. 1:**
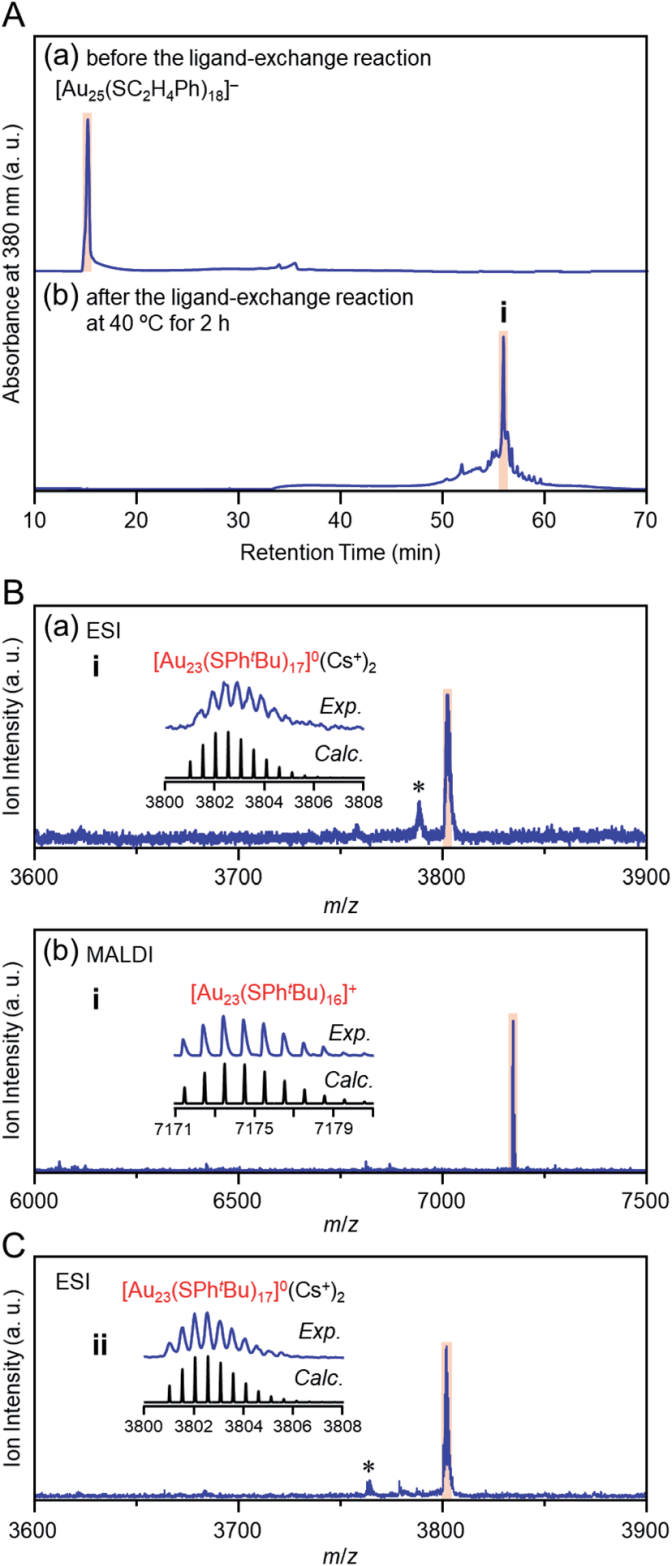
(A) RP-HPLC chromatogram (a) before and (b) after the reaction between [Au_25_(SC_2_H_4_Ph)_18_]^−^ and ^*t*^BuPhSH at 40 °C for 2 h. (B) Positive-ion (a) ESI and (b) MALDI mass spectra of the main product (**i**). (C) Positive-ion ESI mass spectrum of product **ii** (Fig. S3A(b)†). In the ESI-MS, Cs^+^ was added as a cation source. In (B) and (C), the insets compare the isotope pattern between the experimental and simulated spectra. In (B) and (C), the peak with asterisk (*) was assigned to [Au_23_(SC_2_H_4_Ph)(SPh^*t*^Bu)_16_]^0^(Cs^+^)_2_ and [Au_23_(SC_4_H_9_)(SPh^*t*^Bu)_16_]^0^(Cs^+^)_2_, respectively.

Fig. 1A(a) and (b) show reversed-phase high-performance liquid chromatography (RP-HPLC)^39–42^ chromatograms of the precursor [Au_25_(SC_2_H_4_Ph)_18_]^−^ (Fig. S1†) and the product, respectively. The chromatogram of the sample before the reaction (Fig. 1A(a)) showed a sharp peak at the retention time of [Au_25_(SC_2_H_4_Ph)_18_]^−^ (16.0 min). In the product chromatogram (Fig. 1A(b)), the main peak (**i**) was observed at a retention time of 55.6 min, which differs from that of [Au_25_(SC_2_H_4_Ph)_18_]^−^ and [Au_28_(SPh^*t*^Bu)_20_]^0^ (59.7 min, Fig. S2†) (Table S1†).

We isolated product **i** and measured its electrospray ionization (ESI) mass spectrum to determine the chemical composition of product **i**. In the ESI mass spectrometry (MS), cesium ions (Cs^+^) were added to the NC solution as an ionization source.^43^Fig. 1B(a) shows the ESI mass spectrum of product **i**. In the mass spectrum, a strong peak was observed at the position (*m*/*z* = 3802.6) that was assignable to [Au_23_(SPh^*t*^Bu)_17_]^0^(Cs^+^)_2_ (*M*_w_ = 7605.3). This result suggests that product **i** is neutral [Au_23_(SPh^*t*^Bu)_17_]^0^. We also measured the matrix-assisted laser desorption/ionization (MALDI) mass spectrum of product **i**. A strong peak was observed at the position (*m*/*z* = 7173.4) that was assignable to [Au_23_(SPh^*t*^Bu)_16_]^+^ (Fig. 1B(b)). In the MALDI-MS of Au_*n*_(SPh^*t*^Bu)_*m*_, one SPh^*t*^Bu is typically released from Au_*n*_(SPh^*t*^Bu)_*m*_ NCs by laser irradiation.^35,44,45^ The main peak was observed at a position of [Au_23_(SPh^*t*^Bu)_16_]^+^ in the MALDI mass spectrum of [Au_23_(SPh^*t*^Bu)_17_]^0^ because a similar release of one SPh^*t*^Bu was caused by laser irradiation. We also synthesized [Au_25_(SC_4_H_9_)_18_]^0^ with SC_4_H_9_ (1-butanethiolate, Scheme S1(c) and Fig. S3A(a)†) as a ligand and reacted it with ^*t*^BuPhSH. In the ESI (Fig. 1C) and MALDI mass spectra (Fig. S3B†) of the main product (**ii**, Fig. S3A(b)†), a strong peak was observed in the same positions as the product **i** (*m*/*z* = 3802.6 and 7173.4, respectively). Therefore, the main product contains only SPh^*t*^Bu as a ligand. These results confirmed the chemical composition of the main product (**i**) as [Au_23_(SPh^*t*^Bu)_17_]^0^.

**Fig. 2:**
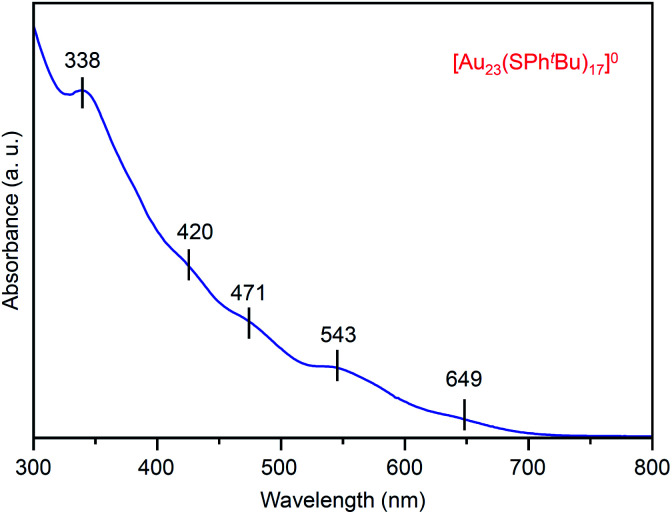
UV-vis optical absorption spectrum of product **i** ([Au_23_(SPh^*t*^Bu)_17_]^0^).

Fig. 2 shows the ultraviolet-visible (UV-vis) optical absorption spectrum of the obtained [Au_23_(SPh^*t*^Bu)_17_]^0^ (**i**). In the spectrum, peak structures appeared at ∼650, 545, 470, 420 and 340 nm. These peak positions are different from those in the spectrum of [Au_25_(SC_2_H_4_Ph)_18_]^−^ (∼675, 445, 395 and 320 nm; Fig. S1(c)†),^37,46^ which indicates that [Au_23_(SPh^*t*^Bu)_17_]^0^ has a different electronic structure from its precursor, [Au_25_(SC_2_H_4_Ph)_18_]^−^. For the geometric structure of the obtained [Au_23_(SPh^*t*^Bu)_17_]^0^, the structure in Fig. S4† may be one of the candidates based on the DFT calculations results^47^ on [Au_23_(SCH_3_)_17_]^0^ (SCH_3_ = methanethiolate). Although we attempted to crystallize [Au_23_(SPh^*t*^Bu)_17_]^0^ for one year to demonstrate experimentally the geometric structure by single-crystal X-ray diffraction (SC-XRD),^28,48,49^ we could not obtain good-quality crystals. There might be the structural isomers in [Au_23_(SPh^*t*^Bu)_17_]^0^, leading to the difficulty in the crystallization. It is expected that the geometric structure of [Au_23_(SPh^*t*^Bu)_17_]^0^ will be determined by DFT calculations of NC containing ligands (SPh^*t*^Bu) instead of SCH_3_ (Fig. S5†)^50–52^ in future.

**Fig. 3:**
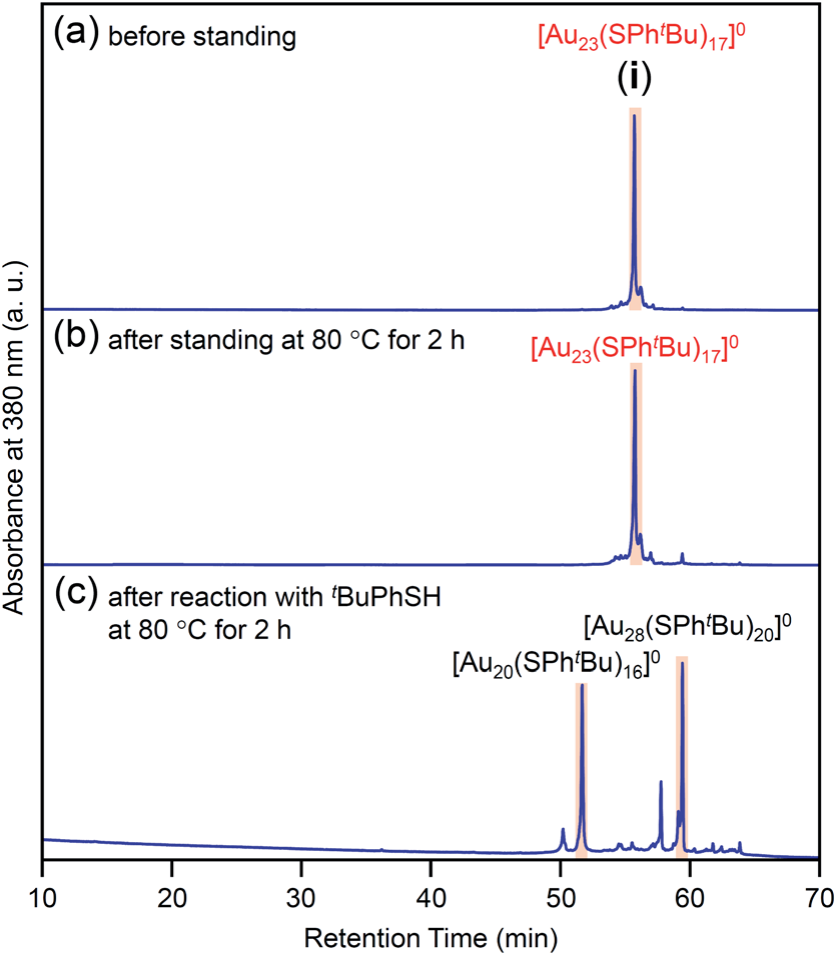
Comparison of RP-HPLC chromatogram of product **i** (a) before and (b) after soaking in toluene solution at 80 °C for 2 h. (c) Chromatogram of products obtained by reacting [Au_23_(SPh^*t*^Bu)_17_]^0^ (**i**) with ^*t*^BuPhSH in toluene solution at 80 °C (reaction time, 2 h). In (c), the main peaks at 51.6 and 59.7 min are attributed to the reported [Au_20_(SPh^*t*^Bu)_16_]^0^ (Fig. S10†) and [Au_28_(SPh^*t*^Bu)_20_]^0^ (Fig. S2†), respectively. The sub peaks at 50.2 and 57.6 min are attributed to [Au_19_(SPh^*t*^Bu)_15_]^0^ and [Au_29_(SPh^*t*^Bu)_17_]^0^, respectively (Fig. S10†).

In this way, we obtained [Au_23_(SPh^*t*^Bu)_17_]^0^ in a high proportion by the ligand-exchange reaction at 40 °C with a ratio of [^*t*^BuPhSH]/[SC_2_H_4_Ph] ≅ 100 (Scheme 1(d)). The [Au_23_(SPh^*t*^Bu)_17_]^0^ hardly decomposed even when left in a toluene solution at temperatures up to 80 °C (Fig. 3(a), (b) and S6†). Therefore, [Au_23_(SPh^*t*^Bu)_17_]^0^ is stable in solution. Mandal *et al.* have also synthesized a similar NC selectively by ligand exchange using a different NC ([Au_23_(SC_6_H_11_)_16_]^−^, SC_6_H_11_ = cyclohexanethiolate) as a precursor.^53^ They assigned the product as Au_23_(SPh^*t*^Bu)_16_ based on the MALDI-MS results. However, according to Fig. 1B(b) and the optical absorption spectrum of the obtained product, it is assumed that [Au_23_(SPh^*t*^Bu)_17_]^0^ was also synthesized selectively. These results indicate that [Au_23_(SPh^*t*^Bu)_17_]^0^ is a metal NC with a level of stability that allows for selective synthesis.

We also investigated the formation mechanism of the [Au_23_(SPh^*t*^Bu)_17_]^0^. MALDI-^27^ or ESI-MS^45^ has been used as a reaction tracking tool in studies on product formation mechanisms. However, in this ligand-exchange reaction, the reactant ([Au_25_(SC_2_H_4_Ph)_18_]^−^) is a negative ion, whereas the product is neutral. Therefore, it is difficult to ionize reactants and products with the same ionization efficiency using neither negative- or positive-ion mode. In addition, in MALDI-MS, [Au_*n*_(SC_2_H_4_Ph)_*m*−*x*_(SPh^*t*^Bu)_*x*_]^0^ can be ionized nondestructively,^27^ whereas [Au_*n*_(SPh^*t*^Bu)_*m*_]^0^ is observed only as being dissociated (Fig. 1B(b)).^35,44,45,53^ Thus, it is extremely difficult to estimate the abundance distribution of the products by MALDI-MS (Fig. S7†). We found that it is also difficult to estimate the abundance distribution of the products for this reaction system using ESI-MS because the reaction intermediates ([Au_*n*_(SC_2_H_4_Ph)_*m*−*x*_(SPh^*t*^Bu)_*x*_]^0^) dissociate during ESI (Fig. S8†).

In our previous studies, we separated SR-protected metal NCs by RP-HPLC (Scheme S3†) depending on the charge state and ligand combination (Scheme S4†).^30,31,54,55^ The charge-state distribution and abundance distribution of the products can be estimated relatively quantitatively by using such a high-resolution RP-HPLC. In addition, it is also possible to reveal the electronic structure of each separated Au_*n*_(SC_2_H_4_Ph)_*m*−*x*_(SPh^*t*^Bu)_*x*_ NC by a photodiode array (PDA) detector (Scheme S3(a)†) attached to the HPLC equipment. Therefore, we attempted to clarify the chemical composition, abundance distribution, and electronic structure of the products by the combined use of MALDI-MS and RP-HPLC.

**Fig. 4:**
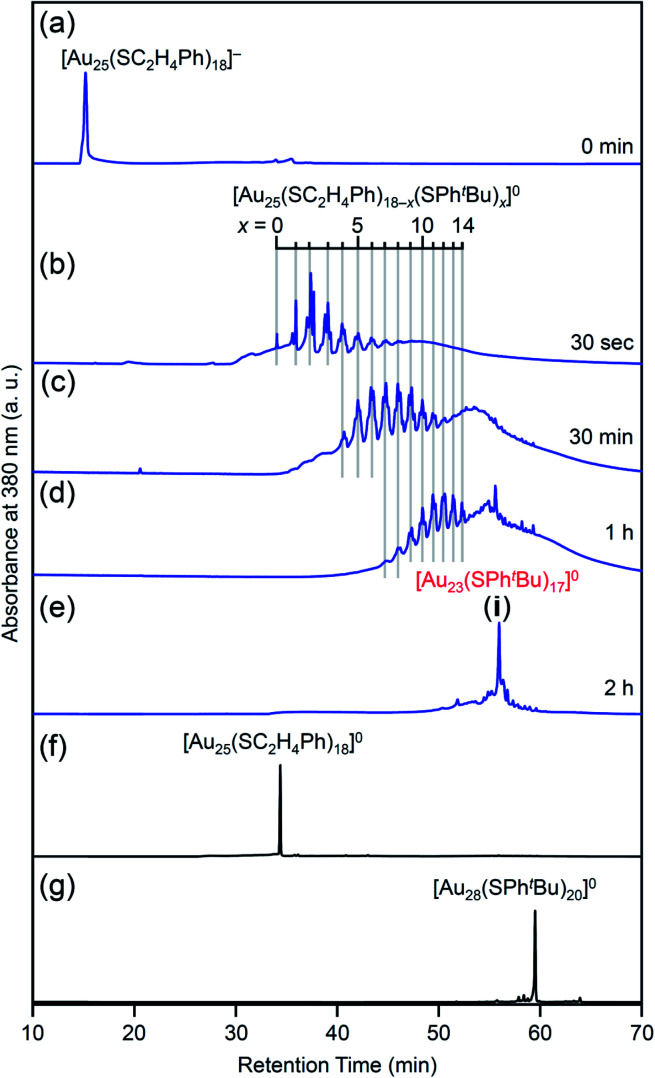
RP-HPLC chromatogram of products obtained by the reaction between [Au_25_(SC_2_H_4_Ph)_18_]^−^ and ^*t*^BuPhSH for (a) 0 min, (b) 30 s, (c) 30 min, (d) 1 h and (e) 2 h (**i**). RP-HPLC chromatogram of (f) [Au_25_(SC_2_H_4_Ph)_18_]^0^ and (g) [Au_28_(SPh^*t*^Bu)_20_]^0^ (Fig. S2†) are shown for comparison. Ligand-exchange products eluted at longer retention times than [Au_25_(SC_2_H_4_Ph)_18_]^0^ because the polarity of SPh^*t*^Bu is lower than that of SC_2_H_4_Ph (Scheme S4†).^54^ The fine peak progression in each major peak is caused by the existence of topological isomers.^55^ The peak attributed to [Au_23_(SPh^*t*^Bu)_17_]^0^ was observed with a weak intensity in the MALDI mass spectra of the products (Fig. S7†) probably because of the different ionization process between [Au_25_(SC_2_H_4_Ph)_18−*x*_(SPh^*t*^Bu)_*x*_]^0^ and [Au_23_(SPh^*t*^Bu)_17_]^0^.^27,35,44,45,53^ This result indicates that the combined use of MALDI-MS and RP-HPLC is important to elucidate the details of the ligand-exchange mechanism while estimating the product distribution for this reaction system.

**Fig. 5:**
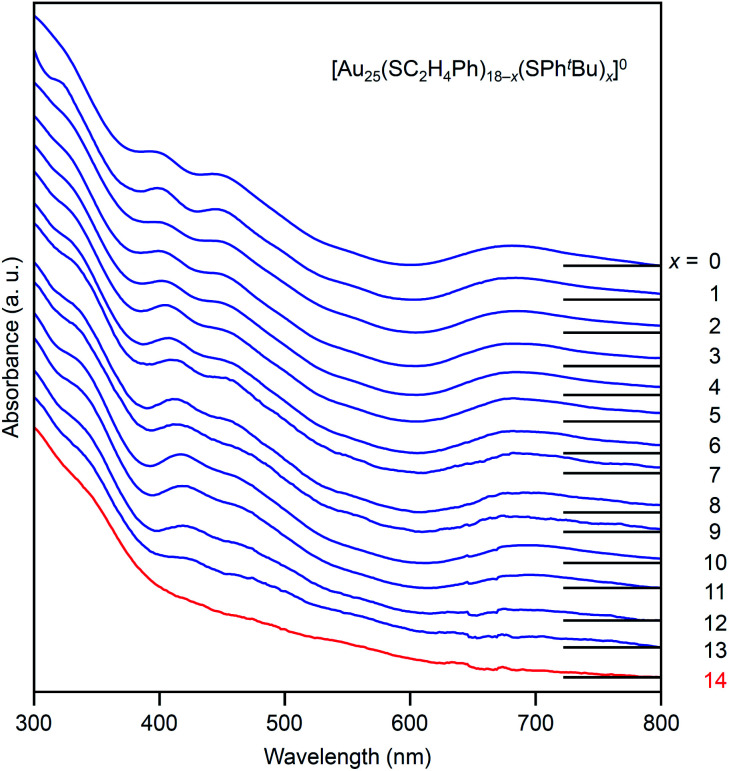
UV-vis optical absorption spectra of a series of [Au_25_(SC_2_H_4_Ph)_18−*x*_(SPh^*t*^Bu)_*x*_]^0^ (*x* = 0–14, Fig. 4) obtained by PDA detector attached to HPLC apparatus (Scheme S3(a)†). These optical absorption spectra are not obtained for the products with a distribution in chemical composition, which has often been reported in the literature,^45^ but for each chemical-composition product that is separated by RP-HPLC (Fig. 4). The spectral features at *x* = 0–13, disappear at *x* = 14, which means that structural deformation starts to occur at *x* = 14.

Fig. 4 shows the RP-HPLC chromatograms of the products at each reaction time (Table S1†). These chromatograms and the MALDI mass spectra of the products at each reaction time (Fig. S7†) show the following four facts for the reaction between [Au_25_(SC_2_H_4_Ph)_18_]^−^ and ^*t*^BuPhSH (Fig. S9†): (1) first, [Au_25_(SC_2_H_4_Ph)_18_]^−^ is oxidized to [Au_25_(SC_2_H_4_Ph)_18_]^0^, (2) then, SC_2_H_4_Ph is exchanged with SPh^*t*^Bu, (3) these exchanges form [Au_25_(SC_2_H_4_Ph)_18−*x*_(SPh^*t*^Bu)_*x*_]^0^ (*x* = 0–14), (4) little [Au_25_(SC_2_H_4_Ph)_18−*x*_(SPh^*t*^Bu)_*x*_]^0^ (*x* ≥ 15) are produced. In addition, the optical absorption spectra (Fig. 5) of a series of [Au_*n*_(SC_2_H_4_Ph)_*m*−*x*_(SPh^*t*^Bu)_*x*_]^0^ (*x* = 0–14) showed that (5) a significant change occurs in the electronic structure of [Au_*n*_(SC_2_H_4_Ph)_*m*−*x*_(SPh^*t*^Bu)_*x*_]^0^ during the increase of *x* from 13 to 14. Based on these five facts, (1) when the number of exchanged ligands reaches 14, the framework of the product begins to distort, and (2) when the number of exchanged ligands increases above 15, the product cannot maintain its framework and changes its structure to [Au_23_(SPh^*t*^Bu)_17_]^0^ which can include SPh^*t*^Bu in a higher ratio (Scheme 2(a)). It is considered that [Au_23_(SPh^*t*^Bu)_17_]^0^ obtained in this study was synthesized selectively by such a reaction mechanism.

We reacted [Au_23_(SPh^*t*^Bu)_17_]^0^ with ^*t*^BuPhSH in toluene solution at 80 °C for 2 h. [Au_23_(SPh^*t*^Bu)_17_]^0^ disappeared and [Au_20_(SPh^*t*^Bu)_16_]^0^ (Fig. S10†) and [Au_28_(SPh^*t*^Bu)_20_]^0^ (Fig. S2†) were produced (Fig. 3c). Therefore, (1) [Au_23_(SPh^*t*^Bu)_17_]^0^ reacts easily with ^*t*^BuPhSH, and (2) such reactions produce [Au_20_(SPh^*t*^Bu)_16_]^0^ and [Au_28_(SPh^*t*^Bu)_20_]^0^ (Scheme 2(b)). It appears that [Au_20_(SPh^*t*^Bu)_16_]^0^ and [Au_28_(SPh^*t*^Bu)_20_]^0^ (Fig. S11†) were synthesized in previous LEIST experiments (Scheme 1(a) and (b))^25,26,53^ because a similar reaction occurred in the solution.

### Reaction between [Au_24_Pd(SC_2_H_4_Ph)_18_]^0^ and ^*t*^BuPhSH

Our previous studies^56^ have revealed that the reaction of [Au_24_Pd(SC_12_H_25_)_18_]^0^ (SC_12_H_25_ = 1-dodecanethiolate) with RSH (RSH = 1-hexanethiol, 1-octanethiol, 2-phenylethanethiol, *etc.*) proceeds at a faster speed than that of [Au_25_(SC_2_H_4_Ph)_18_]^−^ with RSH. Accordingly, we conducted the reaction between [Au_24_Pd(SC_2_H_4_Ph)_18_]^0^ and ^*t*^BuPhSH at 25 °C to decrease the reaction speed and thereby create the novel metal NC.

**Fig. 6:**
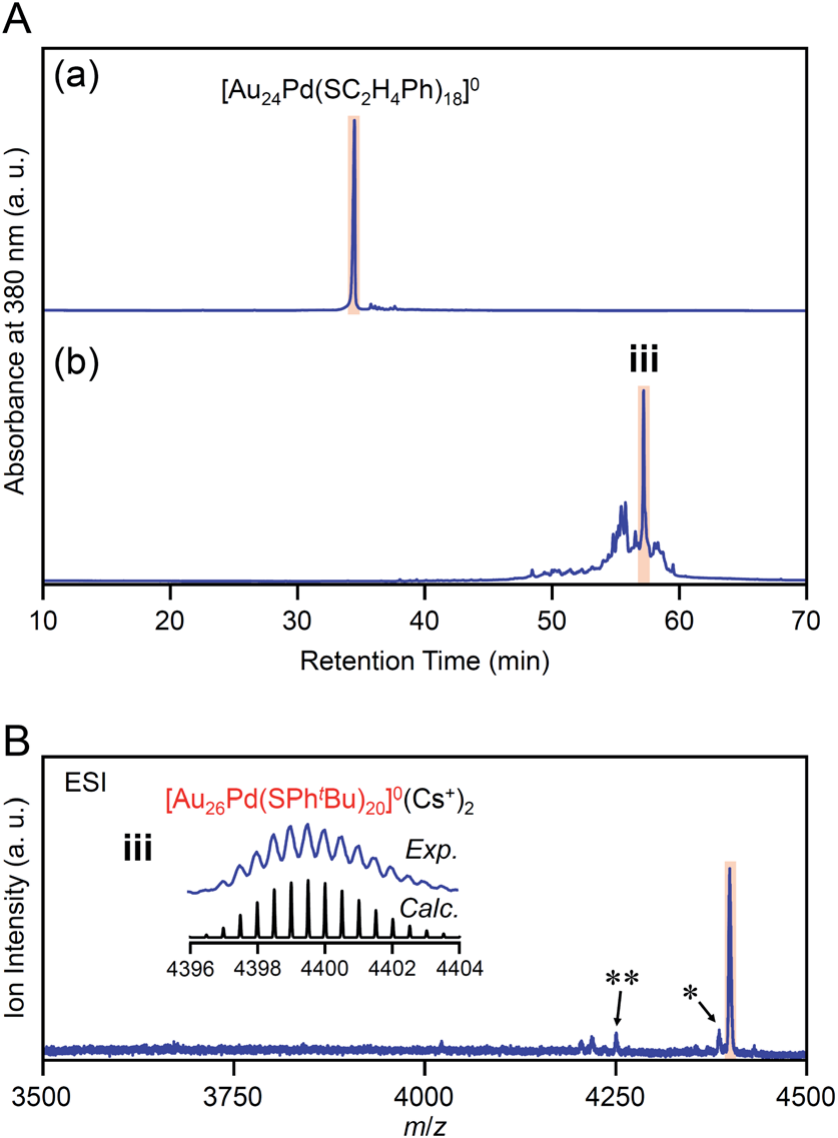
(A) RP-HPLC chromatogram of (a) before and (b) after the reaction between [Au_24_Pd(SC_2_H_4_Ph)_18_]^0^ and ^*t*^BuPhSH for 8 h. (B) Positive-ion ESI mass spectrum of the main products obtained by the reaction between [Au_24_Pd(SC_2_H_4_Ph)_18_]^0^ and ^*t*^BuPhSH (**iii**). The inset compares the isotope pattern between the experimental and simulated spectra. Minor peaks with (*) and (**) are assigned to [Au_26_Pd(SC_2_H_4_Ph)(SPh^*t*^Bu)_19_]^0^(Cs^+^)_2_ and [Au_27_Pd(SPh^*t*^Bu)_17_]^0^(Cs^+^)_2_, respectively.

In the experiment, ^*t*^BuPhSH was added to a toluene solution of [Au_24_Pd(SC_2_H_4_Ph)_18_]^0^ at a ratio of [^*t*^BuPhSH]/[SC_2_H_4_Ph] ≅ 250, and the solution was stirred at 25 °C for 8 h (Scheme 1(e)). Fig. 6A(a) and (b) shows the RP-HPLC chromatogram of samples before (Fig. S12†) and after the reaction, respectively. The chromatogram of the sample before the reaction showed a sharp peak at the retention time of [Au_24_Pd(SC_2_H_4_Ph)_18_]^0^ (34.7 min). The chromatogram of the sample after the reaction (namely, the product) showed a sharp peak (**iii**) at a retention time of 57.4 min.

We isolated product **iii** and measured its ESI mass spectrum to determine the chemical composition of product **iii**. In this MS, Cs^+^ was also added to the NC solution as an ionization source. Fig. 6B shows the ESI mass spectrum of product **iii** after isolation. In the mass spectrum, a strong peak was observed at the position (*m*/*z* = 4399.16) that is attributed to [Au_26_Pd(SPh^*t*^Bu)_20_]^0^(Cs^+^)_2_ (*M*_w_ = 8798.32). This result suggests that product **iii** is neutral [Au_26_Pd(SPh^*t*^Bu)_20_]^0^. We also synthesized [Au_24_Pd(SC_4_H_9_)_18_]^0^ (Fig. S13(a)–(c)†), which has different ligands from [Au_24_Pd(SC_2_H_4_Ph)_18_]^0^, and reacted it with ^*t*^BuPhSH. In the ESI mass spectrum of the main product (**iv**, Fig. S14†), a strong peak was observed at the same position (*m*/*z* = 4399.16) as that of product **iii**. These results indicate that the main product contains only SPh^*t*^Bu as a ligand, which supports the above interpretation that product **iii** is [Au_26_Pd(SPh^*t*^Bu)_20_]^0^. Inductively coupled plasma MS confirmed that product **iii** contains Pd (Au : Pd = 26 : 1.17). Similar result was also obtained by X-ray photoelectron spectroscopy (Fig. S15†).

**Fig. 7:**
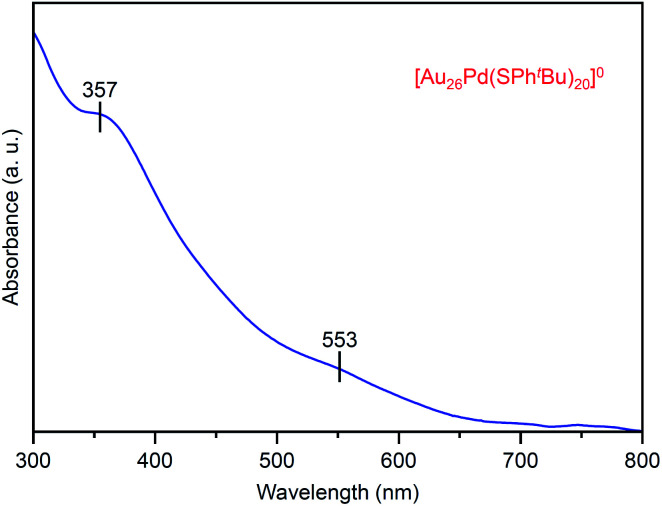
UV-vis optical absorption spectrum of product **iii** ([Au_26_Pd(SPh^*t*^Bu)_20_]^0^).

Fig. 7 shows the optical absorption spectrum of [Au_26_Pd(SPh^*t*^Bu)_20_]^0^. In the spectrum, peaks were observed at ∼550 and 360 nm. These peak positions are different from those observed in the spectrum of [Au_24_Pd(SC_2_H_4_Ph)_18_]^0^ (∼950, 650, 470 and 370 nm; Fig. S12(c)†). This result indicates that the electronic structure of [Au_26_Pd(SPh^*t*^Bu)_20_]^0^ is different from that of [Au_24_Pd(SC_2_H_4_Ph)_18_]^0^. The X-ray absorption fine structure analysis (Fig. S16(a)†) revealed that Au in [Au_26_Pd(SPh^*t*^Bu)_20_]^0^ is a little reduced than Au in mono-Au [Au_28_(SPh^*t*^Bu)_20_]^0^. As for the geometrical structure, because Pd is typically located at the center of the metal core in alloy NCs that consist of Au and Pd,^30,57–61^ it is reasonable to assume that Pd is also located at the center of the metal core in [Au_26_Pd(SPh^*t*^Bu)_20_]^0^ that was obtained in this study (Fig. S17†). Unfortunately, crystallization of [Au_26_Pd(SPh^*t*^Bu)_20_]^0^ was not achieved in this study regardless of the repeated trials for one year. However, the Fourier transform extended X-ray absorption fine structure spectrum demonstrates that Au–S and Au–Au bonds also exist in [Au_26_Pd(SPh^*t*^Bu)_20_]^0^ as well as in [Au_28_(SPh^*t*^Bu)_20_]^0^ (Fig. S16(b) and (c)†). The detail of the geometrical structure of [Au_26_Pd(SPh^*t*^Bu)_20_]^0^ is expected to be elucidated by SC-XRD or DFT calculations in a future study.

In this way, we have obtained [Au_26_Pd(SPh^*t*^Bu)_20_]^0^ by the reaction between [Au_24_Pd(SC_2_H_4_Ph)_18_]^0^ and ^*t*^BuPhSH. The obtained [Au_26_Pd(SPh^*t*^Bu)_20_]^0^ showed a high stability in toluene solution at 25 °C (Fig. S18†). However, [Au_26_Pd(SPh^*t*^Bu)_20_]^0^ was not synthesized by the co-reduction of Au and Pd salts in the presence of ^*t*^BuPhSH. These results indicate that it is extremely important to use a ligand-exchange reaction to synthesize [Au_26_Pd(SPh^*t*^Bu)_20_]^0^.

**Fig. 8:**
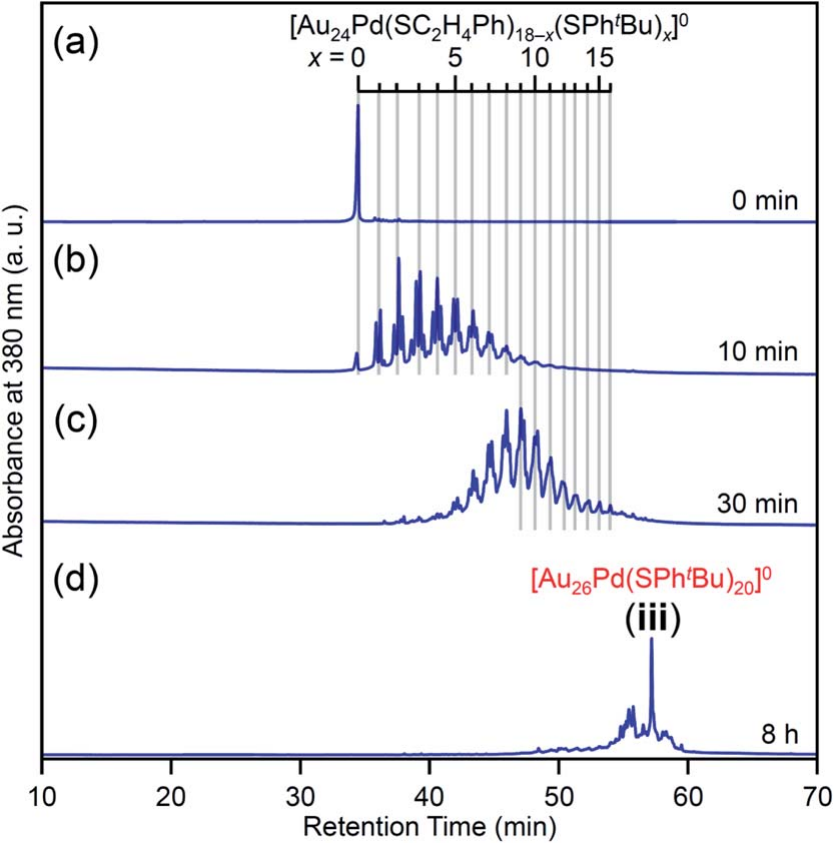
RP-HPLC chromatogram of products obtained by the reaction between [Au_24_Pd(SC_2_H_4_Ph)_18_]^0^ and ^*t*^BuPhSH for (a) 0 min, (b) 10 min, (c) 30 min and (d) 8 h (**iii**). Ligand-exchange products eluted at longer retention times than [Au_24_Pd(SC_2_H_4_Ph)_18_]^0^ because the polarity of SPh^*t*^Bu is lower than that of SC_2_H_4_Ph (Scheme S4†).^54^ The fine peak progression in each major peak is caused by the existence of topological isomers.^55^

We investigated the chemical composition, abundance distribution, and electronic structure of the products for this reaction system by MALDI-MS (Fig. S19†), RP-HPLC (Fig. 8 and Table S2†) and optical absorption spectroscopy (Fig. S20†). The following facts were revealed for the reaction between [Au_24_Pd(SC_2_H_4_Ph)_18_]^0^ and ^*t*^BuPhSH (Fig. S21†): (1) only the ligand-exchange reaction proceeds while the number of metal atoms, ligands and the electronic structure of [Au_24_Pd(SC_2_H_4_Ph)_18_]^0^ is maintained (Fig. S20†) until the number of exchanged ligands reaches 16, (2) [Au_24_Pd(SC_2_H_4_Ph)_1_(SPh^*t*^Bu)_17_]^0^ with 17 exchanged ligands is scarcely obtained (Fig. 8 and S19†). As mentioned above, when the central atom of [Au_25_(SC_2_H_4_Ph)_18_]^−^ was replaced by a Pt atom ([Au_24_Pt(SC_2_H_4_Ph)_18_]^0^), the framework of NC strengthens, and thereby [Au_24_Pt(SPh^*t*^Bu)_18_]^0^, in which all ligands are exchanged with SPh^*t*^Bu, was also obtained.^35^ Although the substitution of the central atom of Au_25_(SR)_18_ with a Pd atom improves the stability against decomposition in solution compared with Au_25_(SR)_18_,^30^ based on the interaction energy^36^ between the central atom (Au, Pd, or Pt) and the surrounding Au_24_(SR)_18_ structure, [Au_24_Pd(SC_2_H_4_Ph)_18_]^0^ is interpreted to have a framework that is not as strong as [Au_24_Pt(SC_2_H_4_Ph)_18_]^0^. The strongness of this structure is considered the reason why [Au_24_Pd(SC_2_H_4_Ph)_18−*x*_(SPh^*t*^Bu)_*x*_]^0^ cannot maintain its framework structure when more than 16 SC_2_H_4_Ph are exchanged with SPh^*t*^Bu, and changes its structure to [Au_26_Pd(SPh^*t*^Bu)_20_]^0^, which can contain more SPh^*t*^Bu (Scheme 3(a)), unlike [Au_24_Pt(SC_2_H_4_Ph)_18−*x*_(SPh^*t*^Bu)_*x*_]^0^.

We then left the obtained [Au_26_Pd(SPh^*t*^Bu)_20_]^0^ in toluene solution at 60 °C for 14 h. As a result, [Au_28_(SPh^*t*^Bu)_20_]^0^, which contains only Au as a metal element, was formed as the main product (Fig. S22†). Although the geometric structure of [Au_28_(SPh^*t*^Bu)_20_]^0^ (Scheme S2(b)†) has been revealed to contain two central Au atoms,^25^ Au_27_Pd(SPh^*t*^Bu)_20_ and Au_26_Pd_2_(SPh^*t*^Bu)_20_, in which one or two central atoms was replaced by a Pd atom, respectively, were not produced in our experiments. In icosahedral structures, such as the metal core of [Au_25_(SC_2_H_4_Ph)_18_]^−^ (Scheme S2(a)†), the bond length between the 12 surface atoms is extended by ∼5% compared with the distance between the central and surface atoms (Fig. S23(a)†). Thus, the central atom of the icosahedral structure is easily replaced by an atom with a smaller atomic radius.^62^ The metal core of [Au_28_(SPh^*t*^Bu)_20_]^0^ has a geometric structure in which two cubic octahedra are connected. In this structure, no large difference exists between the bond lengths of the central–surface atoms and those of the surface–surface atoms (Fig. S23(b)†). For such a geometric structure, formation of a core–shell structure with Pd as the core and Au as the shell typically dose not occur. Although the chemical composition of the final product is affected by the reaction path,^63,64^ this geometric factor appears to be largely related to the fact that Au_27_Pd(SPh^*t*^Bu)_20_ and Au_26_Pd_2_(SPh^*t*^Bu)_20_ were not formed in our experiments.

### Reaction between [Au_24_Pt(SC_2_H_4_Ph)_18_]^0^ and ^*t*^BuPhSH

**Fig. 9:**
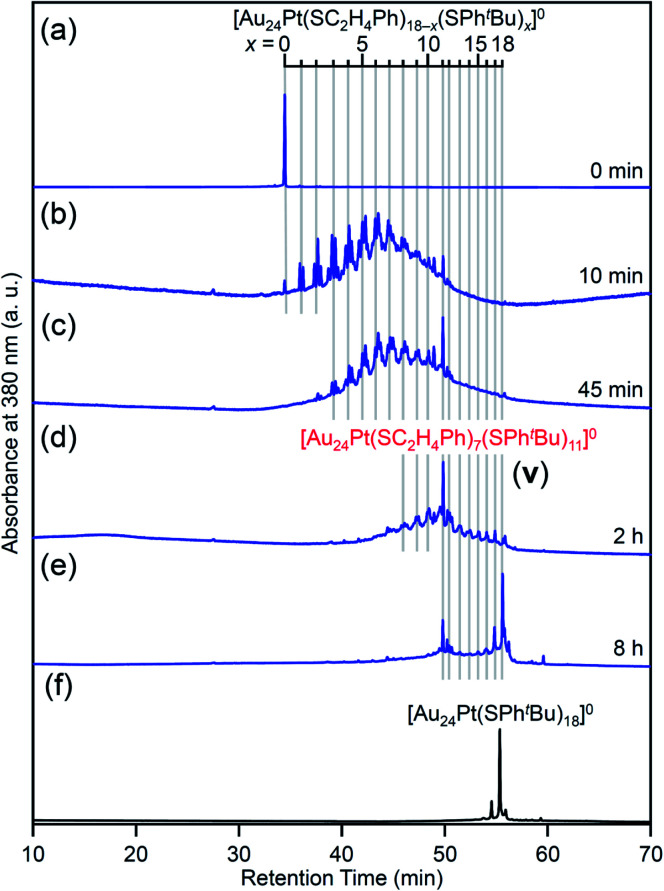
RP-HPLC chromatogram of products obtained by the reaction between [Au_24_Pt(SC_2_H_4_Ph)_18_]^0^ and ^*t*^BuPhSH: (a) 0 min, (b) 10 min, (c) 45 min, (d) 2 h and (e) 8 h with that of (f) [Au_24_Pt(SPh^*t*^Bu)_18_]^0^ (Fig. S24†). Ligand-exchange products eluted at longer retention times than [Au_24_Pt(SC_2_H_4_Ph)_18_]^0^ because the polarity of SPh^*t*^Bu is lower than that of SC_2_H_4_Ph.^54^ Fine peak progression in each major peak is caused by the existence of topological isomers.^55^ The retention time of [Au_24_Pt(SC_2_H_4_Ph)_18−*x*_(SPh^*t*^Bu)_*x*_]^0^ (*x* = 0–18, Table S3†) becomes irregular around *x* = 11, which shows the specificity of [Au_24_Pt(SC_2_H_4_Ph)_7_(SPh^*t*^Bu)_11_]^0^.

**Fig. 10:**
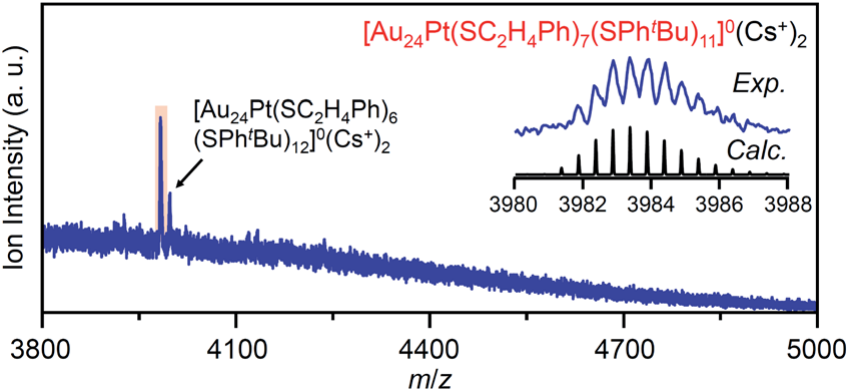
Positive-ion ESI mass spectra of main products (**v**) obtained by the reaction between [Au_24_Pt(SC_2_H_4_Ph)_18_]^0^ and ^*t*^BuPhSH for 2 h. The inset compares the isotope pattern between the experimental and simulated spectra. In this sample, [Au_24_Pt(SC_2_H_4_Ph)_6_(SPh^*t*^Bu)_12_]^0^ was also included as a minor product.

**Scheme 4:**
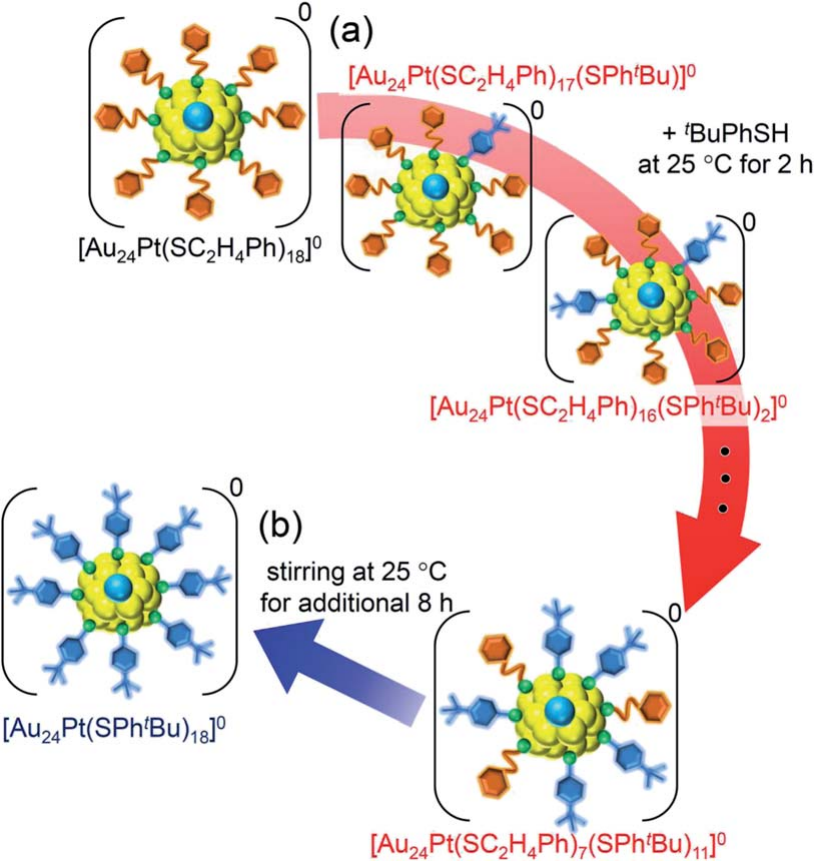
Schematic illustration of the transformation from (a) [Au_24_Pt(SC_2_H_4_Ph)_18_]^0^ to [Au_24_Pt(SC_2_H_4_Ph)_7_(SPh^*t*^Bu)_11_]^0^ (**v**) and (b) [Au_24_Pt(SC_2_H_4_Ph)_7_(SPh^*t*^Bu)_11_]^0^ (**v**) to [Au_24_Pt(SPh^*t*^Bu)_18_]^0^.

For this reaction system, our previous studies have shown that [Au_24_Pt(SPh^*t*^Bu)_18_]^0^, in which all ligands are replaced by SPh^*t*^Bu, can be synthesized selectively after reaction for 10 h (Scheme 1(f)).^35^ In the present study, we found that a specific reaction intermediate ([Au_24_Pt(SC_2_H_4_Ph)_7_(SPh^*t*^Bu)_11_]^0^) can be obtained in a high proportion (Fig. 9 and 10) when the ligand-exchange reaction was stopped at 2 h (Scheme 4).

In the experiment, ^*t*^BuPhSH was added to a toluene solution of [Au_24_Pt(SC_2_H_4_Ph)_18_]^0^ (Fig. S24†) at a ratio of [^*t*^BuPhSH]/[SC_2_H_4_Ph] ≅ 250, and the solution was stirred at 25 °C. The chromatogram of the sample after 2 h of reaction showed a strong peak (**v**) at the retention time of 49.8 min (Fig. 9d and Table S3†). ESI-MS of the isolated product **v** revealed that product **v** had the chemical composition of [Au_24_Pt(SC_2_H_4_Ph)_7_(SPh^*t*^Bu)_11_]^0^ (Fig. 10).

In this way, when the reaction between [Au_24_Pt(SPh^*t*^Bu)_18_]^0^ and ^*t*^BuPhSH is stopped in a short time, [Au_24_Pt(SC_2_H_4_Ph)_7_(SPh^*t*^Bu)_11_]^0^ can be obtained in a high proportion (Scheme 4(a)). It appears that [Au_24_Pt(SC_2_H_4_Ph)_7_(SPh^*t*^Bu)_11_]^0^ is thermodynamically stabilized because steric repulsion between surface ligands is lowest and/or the C–H⋯π interaction (Fig. S25†)^35^ is highest at this chemical composition, which leads to the formation of [Au_24_Pt(SC_2_H_4_Ph)_7_(SPh^*t*^Bu)_11_]^0^ in a high proportion (Fig. S26†). In the reactions of [Au_25_(SC_2_H_4_Ph)_18_]^−^ (Fig. 4 and S7†) or [Au_24_Pd(SC_2_H_4_Ph)_18_]^0^ (Fig. 8 and S17†) with ^*t*^BuPhSH, [Au_25_(SC_2_H_4_Ph)_7_(SPh^*t*^Bu)_11_]^0^ and [Au_24_Pd(SC_2_H_4_Ph)_7_(SPh^*t*^Bu)_11_]^0^ were not synthesized in a high proportion. A comparison of these three NCs shows slight differences in the geometric structure (Fig. S27†)^37,38,57^ and the strength of the framework structure.^36^ It is assumed that these factors are related to the selective synthesis of NC with 11 exchanged ligands only in the case of the reaction between [Au_24_Pt(SPh^*t*^Bu)_18−*x*_(SPh^*t*^Bu)_*x*_]^0^ and ^*t*^BuPhSH.

**Fig. 11:**
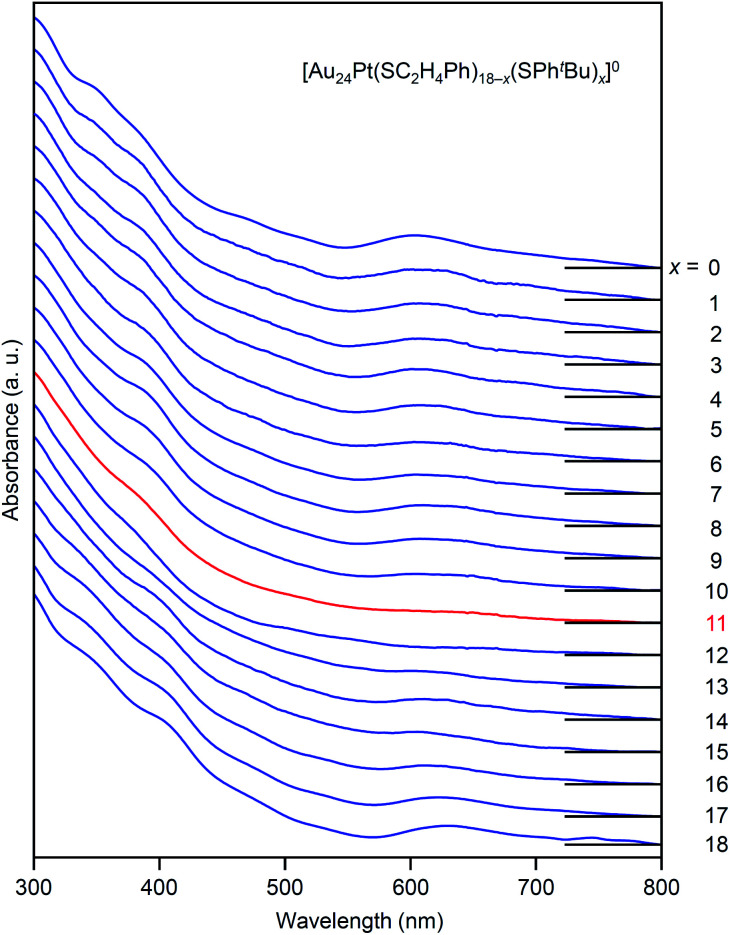
Comparison of UV-vis optical absorption spectra in a series of [Au_24_Pt(SC_2_H_4_Ph)_18−*x*_(SPh^*t*^Bu)_*x*_]^0^ (*x* = 0–18). These optical absorption spectra are not obtained for products with a distribution in chemical composition, which has often been reported in the literature,^45^ but are obtained for each chemical-composition product separated by RP-HPLC (Fig. 9). The spectral features disappear around *x* = 11 and then reappear at *x* ≥ 13, which shows the specificity of [Au_24_Pt(SC_2_H_4_Ph)_7_(SPh^*t*^Bu)_11_]^0^ in a series of [Au_24_Pt(SC_2_H_4_Ph)_18−*x*_(SPh^*t*^Bu)_*x*_]^0^ (*x* = 0–18).

The specificity of [Au_24_Pt(SC_2_H_4_Ph)_7_(SPh^*t*^Bu)_11_]^0^ was also observed in the optical absorption spectrum. Fig. 11 shows the optical absorption spectra of each [Au_24_Pt(SC_2_H_4_Ph)_18−*x*_(SPh^*t*^Bu)_*x*_]^0^ (*x* = 0–18) separated by RP-HPLC. The peak near 600 nm is attributed to a transition from the highest occupied molecular orbital to the lowest unoccupied molecular orbital (LUMO)+1.^65^ Because LUMO+1 involves orbitals that are derived from sulfur in addition to metal core orbitals,^66^ a replacement of the ligand in [Au_24_Pt(SC_2_H_4_Ph)_18_]^0^ with SPh^*t*^Bu ([Au_24_Pt(SPh^*t*^Bu)_18_]^0^) shifts this peak to the longer wavelength side.^35^Fig. 11 shows that (1) these shifts occur continuously with the number of exchanged ligands up to *x* = 10, (2) the above-mentioned peaks become less apparent when *x* reaches 11 and (3) the peaks appear again at *x* ≥ 13, and then shift continuously to the longer wavelength side with the number of exchanged ligands. This fact also implies that [Au_24_Pt(SC_2_H_4_Ph)_7_(SPh^*t*^Bu)_11_]^0^ is a special NC in a series of [Au_24_Pt(SC_2_H_4_Ph)_18−*x*_(SPh^*t*^Bu)_*x*_]^0^ (*x* = 0–18). The origin of this specificity of [Au_24_Pt(SC_2_H_4_Ph)_7_(SPh^*t*^Bu)_11_]^0^ is expected to be elucidated by SC-XRD and DFT calculations in the future study.

## Conclusions

**Fig. 12:**
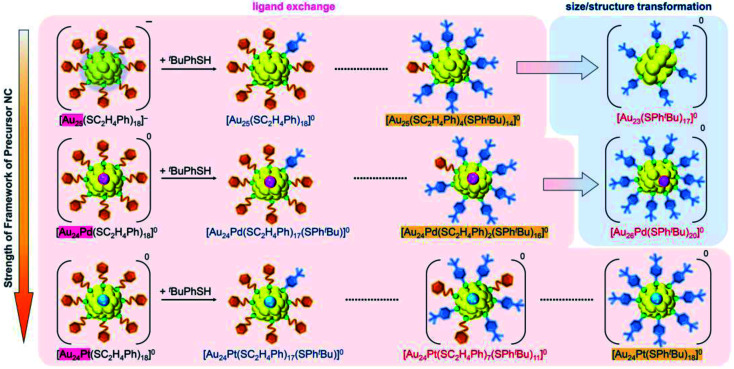
Relation between the strength of the framework structure of the precursor NC and the number of changeable ligands.

In this study, the reaction of [Au_25_(SC_2_H_4_Ph)_18_]^−^ with ^*t*^BuPhSH was selected as a model reaction, and the amount of thiol to be reacted, the central atom of the precursor NCs, or the reaction time were changed from previous studies to create new atomically precise metal NCs. The following findings were obtained:

(1) When ^*t*^BuPhSH is added to a toluene solution in a ratio of [^*t*^BuPhSH]/[SC_2_H_4_Ph] ≅ 100 and the reaction is allowed to proceed for 2 h at 40 °C, [Au_23_(SPh^*t*^Bu)_17_]^0^ is obtained in a high proportion. In such a reaction, the framework of the product begins to distort when the number of exchanged ligands reaches 14. As the ligand exchange proceeds beyond this number, the product cannot maintain its framework structure, and its framework changes to [Au_23_(SPh^*t*^Bu)_17_]^0^. When the obtained [Au_23_(SPh^*t*^Bu)_17_]^0^ is reacted with ^*t*^BuPhSH in toluene solution at 80 °C, [Au_23_(SPh^*t*^Bu)_17_]^0^ changes its framework to the previously reported [Au_20_(SPh^*t*^Bu)_16_]^0^ and [Au_28_(SPh^*t*^Bu)_20_]^0^.

(2) When the central Au atom of [Au_25_(SC_2_H_4_Ph)_18_]^−^ is replaced with a Pd atom ([Au_24_Pd(SC_2_H_4_Ph)_18_]^0^), ^*t*^BuPhSH is added to the toluene solution in a ratio of [^*t*^BuPhSH]/[SC_2_H_4_Ph] ≅ 250, and the reaction is allowed to proceed for 8 h at 25 °C, [Au_26_Pd(SPh^*t*^Bu)_20_]^0^ is obtained in a high proportion. In such a reaction, only ligand exchange occurs with the framework structure retained until the number of the exchanged ligands reaches 16. However, as the ligand exchange proceeds beyond this number, the product is no longer able to maintain the framework, and its framework changes to [Au_26_Pd(SPh^*t*^Bu)_20_]^0^.

(3) When the central Au atom of [Au_25_(SC_2_H_4_Ph)_18_]^−^ is replaced with a Pt atom ([Au_24_Pt(SC_2_H_4_Ph)_18_]^0^), ^*t*^BuPhSH is added to the toluene solution in a ratio of [^*t*^BuPhSH]/[SC_2_H_4_Ph] ≅ 250, and the reaction is allowed to proceed at 25 °C for only 2 h, [Au_24_Pt(SC_2_H_4_Ph)_7_(SPh^*t*^Bu)_11_]^0^ is obtained in a high proportion.

(4) The differences in ligand-exchange reaction mechanisms of the above three reactions are caused mainly by the slight differences in framework strength (Fig. 12) and geometrical structure of the precursor metal NCs (Fig. S27†).

To the best of our knowledge, no report exists on the selective synthesis of these three metal NCs ([Au_23_(SPh^*t*^Bu)_17_]^0^, [Au_26_Pd(SPh^*t*^Bu)_20_]^0^ and [Au_24_Pt(SC_2_H_4_Ph)_7_(SPh^*t*^Bu)_11_]^0^). These results indicate that the reaction pathway in LEIST^67^ and the chemical composition of the final products can be modified by controlling the amount of thiol, the type of central atom and the reaction time, which demonstrates that a variety of atomically precise metal NCs, which are synthesized size-selectively, can be increased compared with current NCs if the ligand-exchange reaction is conducted while changing the reaction conditions and/or the central atoms of the precursor metal NCs from previous studies. We have also elucidated the details of the reaction mechanism in the ligand-exchange reaction by using both MS and RP-HPLC, which was difficult using only MS often used in the literature. In future, it is expected that the details of the reaction mechanisms of various reactions that have not yet been clarified will be revealed by this combined use.

## Data availability

The data supporting the findings of this study are available within the article and in the ESI.†

## Author contributions

Y. Negishi designed the experiments. H. Horihata. and A. Ebina conducted the measurements along with S. Miyajima, M. Nakamoto and A. Ikeda. S. Hossain and T. Kawawaki helped analyze the obtained results. Y. Negishi wrote the manuscript. All authors have approved the final version of the manuscript.

## Conflicts of interest

There are no conflicts to declare.

## Supplementary Material

SC-013-D2SC00423B-s001
