# Supplementary material for: Selective formation of [Au23(SPhtBu)17]0, [Au26Pd(SPhtBu)20]0 and [Au24Pt(SC2H4Ph)7(SPhtBu)11]0 by controlling ligand-exchange reaction
Source: Chem Sci. 2022 Mar 30;13(19):5546–56. doi: 10.1039/d2sc00423b (PMC9116332; doi:10.1039/d2sc00423b)
Supplement: SC-013-D2SC00423B-s001 [file SC-013-D2SC00423B-s001.pdf]

Electronic Supplementary Material (ESI) for Chemical Science.
This journal is © The Royal Society of Chemistry 2022

Supporting Information

Selective formation of $[Au_{23}(SPh^tBu)_{17}]^0$, $[Au_{26}Pd(SPh^tBu)_{20}]^0$ and $[Au_{24}Pt(SC_2H_4Ph)_7(SPh^tBu)_{11}]^0$ by controlling ligand-exchange reaction

Yuichi Negishi,[1,2,*] Hikaru Horihata,[1] Ayano Ebina,[1] Sayuri Miyajima,[1] Mana Nakamoto,[1] Ayaka Ikeda,[1] Tokuhisa Kawawaki,[1,2] and Sakiat Hossain[1]

[1]*Department of Applied Chemistry, Faculty of Science, Tokyo University of Science, Kagurazaka, Shinjuku−ku, Tokyo 162−8601, Japan*

[2] *Research Institute for Science & Technology, Tokyo University of Science, Kagurazaka, Shinjuku−ku, Tokyo 162−8601, Japan*

Corresponding Author E-mail: negishi@rs.tus.ac.jp (Y. Negishi)

S1. Experiments

S1.1. Chemicals

All chemicals were obtained commercially and used without further purification. Hydrogen tetrachloroacurate tetrahydrate ($HAuCl_4{\cdot}4H_2O$) and chloroplatinic acid hexahydrate ($H_2PtCl_6{\cdot}6H_2O$) were purchased from Tanaka Kikinzoku. Sodium tetrahydroborate ($NaBH_4$), palladium(II) acetate ($(CH_3COO)_2Pd$), 1-butanethiol (C_4H_9SH), tetraoctylammonium bromide (TOABr), gold (Au) standard solution (1.00 mg/mL), Pd standard solution (1.00 mg/mL), bismuth (Bi) standard solution (100 ppm) and silica gel (spherical, 63–210 μm) were from FUJIFILM Wako Pure Chemical Co. 2-Phenyethanethiol (PhC_2H_4SH) was from Sigma Aldrich. Tetrahydrofuran (THF), methanol, toluene, hexane, acetonitrile, diethyl ether, acetone, dichloromethane, and hydrogen peroxide (H_2O_2, 30 wt%) were from Kanto Chemical Co., Inc. Silver plate was from the Nilaco Co. 4-*tert*-Butylbenzenethiol (^{t}BuPhSH) and *trans*-2-[3-(4-*tert*-butylphenyl)-2-methyl-2-propenylidene]malononitrile (DCTB) were from Tokyo Chemical Industry. Co., Ltd. Cesium acetate (CH_3COOCs) was NACALAI TESQUE Inc. Pure Milli-Q water (18.2 MΩ cm) was generated with a Merck Millipore Direct 3 UV system.

S1.2. Synthesis

$[Au_{25}(SC_2H_4Ph)_{18}]^-$ and $[Au_{25}(SC_4H_9)_{18}]^-$. $[Au_{25}(SC_2H_4Ph)_{18}]^-$ (SC_2H_4Ph = 2-phenyethanethiolate; Scheme S1(a)) and $[Au_{25}(SC_4H_9)_{18}]^-$ (SC_4H_9 = 1-butanethiolate; Scheme S1(c)) were synthesized by the reported method[1] with a slight modification. First, 0.75 mmol of $HAuCl_4$ and 0.76 mmol of TOABr were dissolved in 25 mL of THF, and then 4.7 mmol of ligand (PhC_2H_4SH or C_4H_9SH) was added to the solution and the solution was stirred for 30 min at 25 °C. After that, 5 mL of 0 °C water containing 8.7 mmol of $NaBH_4$ was added to the solution to reduce the Au salt ($HAuCl_4$) and the solution was stirred for 10 h at 25 °C. After removing the solvent by rotary evaporator, the precipitates were washed with water, then with a mixture of water and methanol, and finally with pure methanol. The NCs were extracted from the precipitates with toluene. The NC solution was dried again, and then $[Au_{25}(SC_2H_4Ph)_{18}]^-$ or $[Au_{25}(SC_4H_9)_{18}]^-$ was extracted from the product with a mixture of acetone and acetonitrile (mixing ratio = 1:4).

$[Au_{24}Pd(SC_2H_4Ph)_{18}]^0$. $[Au_{24}Pd(SC_2H_4Ph)_{18}]^0$ was synthesized by the reported method[2] with a slight modification. First, 0.89 mmol of $HAuCl_4$, 0.47 mmol of TOABr and 0.030 mmol of $(CH_3COO)_2Pd$ were dissolved in 30 mL of THF, and then the solution was stirred for 15 min at room temperature. Then, 2.6 mmol of PhC_2H_4SH was added to the solution and the solution was stirred 10 min at 25 °C. After that, 5 mL of 0 °C water containing 10 mmol of $NaBH_4$ was added to the solution to reduce the metal salts ($HAuCl_4$ and $(CH_3COO)_2Pd$) and the solution was stirred for 5 h at 25 °C. After removing the solvent by rotary evaporator, the precipitates were washed with water, then with a mixture of water and methanol, and finally with pure methanol. The NCs were extracted from the precipitate with toluene. The NCs solution was dried again and

then a mixture of $[Au_{24}Pd(SC_2H_4Ph)_{18}]^0$ and $[Au_{25}(SC_2H_4Ph)_{18}]^-$ was separated from the product by silica gel column chromatography using a mixture of toluene and hexane (mixing ratio = 1:1) as the mobile phase. After the mixture solution was dried, 5 mL of dichloromethane and 5 mL of H_2O_2 were added to the precipitates, and the solution was stirred for 3 h at 25 °C to decompose $[Au_{25}(SC_2H_4Ph)_{18}]^0$. The product was washed three times with water, and then a part of the products was extracted from the precipitates with dichloromethane. The product was purified by silica gel chromatography as described above to obtain highly pure $[Au_{24}Pd(SC_2H_4Ph)_{18}]^0$.

$[Au_{24}Pd(SC_4H_9)_{18}]^0$. $[Au_{24}Pd(SC_4H_9)_{18}]^0$ was synthesized by slightly modifying the synthesis method of $[Au_{24}Pd(SC_2H_4Ph)_{18}]^0$. First, 0.89 mmol of $HAuCl_4$, 0.47 mmol of TOABr and 0.030 mmol of $(CH_3COO)_2Pd$ were dissolved in 30 mL of THF and then the solution was stirred for 15 min at room temperature. After that, 2.6 mmol of C_4H_9SH was added to the solution and the solution was stirred for 10 min at 25 °C. After that, 5 mL of 0 °C water containing 10 mmol of $NaBH_4$ was added to the solution to reduce the metal salts ($HAuCl_4$ and $(CH_3COO)_2Pd$) and the solution was stirred for 5 h at 25 °C. After removing the solvent by rotary evaporator, the precipitates were washed with water, then with a mixture of water and methanol, and finally with pure methanol. The NCs were extracted from the precipitate with toluene. The NCs solution was dried again and then a mixture of $[Au_{24}Pd(SC_4H_9)_{18}]^0$ and $[Au_{25}(SC_4H_9)_{18}]^-$ was separated from the product by silica gel column chromatography using a mixture of toluene and hexane (mixing ratio = 1:5) as the mobile phase. After the mixture solution was dried, 5 mL of dichloromethane and 5 mL of H_2O_2 were added to the precipitates, and the solution was stirred for 3 h at 25 °C to decompose $[Au_{25}(SC_4H_9)_{18}]^0$. The product was washed three times with water, and then a part of the products was extracted from the precipitates with dichloromethane. The product was purified by silica gel chromatography as described above to obtain highly pure $[Au_{24}Pd(SC_4H_9)_{18}]^0$.

$[Au_{24}Pt(SC_2H_4Ph)_{18}]^0$. $[Au_{24}Pt(SC_2H_4Ph)_{18}]^0$ was synthesized by the reported method[3] with a slight modification. First, 0.20 mmol of $HAuCl_4$, 1.2 mmol of TOABr and 0.030 mmol of H_2PtCl_6 were dissolved in 30 mL of THF and then the solution was stirred for 30 min at 25 °C. After that, 2.5 mmol of PhC_2H_4SH was added to the solution and the solution was stirred for 45 min at 25 °C. Then, 5 mL of 0 °C water containing 10 mmol of $NaBH_4$ was added to the solution to reduce the metal salts ($HAuCl_4$ and H_2PtCl_6) and the solution was stirred for 5 h at 25 °C. After removing the solvent by rotary evaporator, the precipitates were washed with water, then with a mixture of water and methanol, and finally with pure methanol. The NCs were extracted from the precipitate with toluene. The NCs solution was dried again and then a mixture of $[Au_{24}Pt(SC_2H_4Ph)_{18}]^0$ and $[Au_{25}(SC_2H_4Ph)_{18}]^-$ was separated from the product by silica gel column chromatography using a mixture of toluene and hexane (mixing ratio = 5:3) as the mobile phase. After the mixture solution was dried, 5 mL of dichloromethane and 5 mL of H_2O_2 were added to the precipitates, and the solution was stirred for 3 h at 25 °C to decompose $[Au_{25}(SC_2H_4Ph)_{18}]^0$. The product was washed three times with water, and then a part of the products was extracted from the precipitates with dichloromethane. The product was purified by silica gel chromatography as described above to obtain highly pure $[Au_{24}Pt(SC_2H_4Ph)_{18}]^0$.

S1.3. Ligand-Exchange Reaction

$[Au_{25}(SC_2H_4Ph)_{18}]^-$. 44.5 mg of $[Au_{25}(SC_2H_4Ph)_{18}]^-$ was dissolved in 1.0 mL of toluene, and then the solution was heated to 40 °C. The ligand-exchange reaction was carried out by adding 200 µL of *ᵗ*BuPhSH to the solution. At as desired time after the initiation of the reaction, 50 µL of the solution was collected from the reaction solution and the product was washed with water, then with a mixture of water and methanol, and finally with pure methanol.

$[Au_{24}Pd(SC_2H_4Ph)_{18}]^0$, $[Au_{24}Pd(C_4H_9)_{18}]^0$ or $[Au_{24}Pt(SC_2H_4Ph)_{18}]^0$. 3 mg of $[Au_{24}Pd(SC_2H_4Ph)_{18}]^0$, $[Au_{24}Pd(SC_4H_9)_{18}]^0$ or $[Au_{24}Pt(SC_2H_4Ph)_{18}]^0$ was dissolved in 5.0 mL of toluene, and then the ligand-exchange reaction was carried out by adding 400 µL of ^{t}BuPhSH to the solution at 25 °C. At as desired time after the initiation of the reaction, 500 µL of the solution was collected from the reaction solution and the product was washed with water, then with a mixture of water and methanol, and finally with pure

methanol.

S1.4. Separation and Isolation of Product Obtained by Ligand-Exchange Reaction

Shimadzu Prominence high performance liquid chromatography (HPLC) system consisting of LC-20AD (2 pumps), SPD-20A (photodiode array (PDA) detector), CTO-20A (column oven) and DGU-20A (on-line degasser) was used for separation and isolation of products (Scheme S3(a)). YMC core-shell ODS column (Meteoric Core C18, 150 mm × 4.6 mm, I.D., 5 μm)[4] was used as the stationary phase. This column has a non-porous structure in the center of the packing material, which suppresses multi-channel and spiral diffusion, diffusion in the axial direction of the column, and mass transfer by diffusion, and thus provides a higher resolution than that of a commonly used all-porous column.[5] The column and detector were aged (stabilized) for sufficient time prior to analysis. Reversed phase (RP) mode[6] was used for the separation. In the experiment, each precursor NC or the product obtained by ligand exchange was first suspended in a mixture of diethyl ether and acetonitrile (mixing ratio = 13:7). Then, 20 μL of the mixture was injected into a selecting valve. The mobile phase was continuously changed from pure acetonitrile to pure diethyl ether by the gradient program shown in Scheme S3(b). The flow rate of the mobile phase was set to 1.0 mL/min, and the optical absorption spectra of the separated products were obtained with PDA detector in the range of 190–800 nm. The chromatogram was obtained from the absorption intensity at 380 nm.

S1.5. Other Characterization

Electrospray ionization (ESI) mass spectrometry (MS) was performed using a reflectron-type time of flight MS system (Bruker, microTOF II). The NCs were dissolved in a mixture of dichloromethane and methanol. For the measurement of neutral NCs, a few drops of 1 mM CH_3COOCs methanol solution were added to the NC solution by Pasteur pipette because a cation source is required. The isotope distribution was calculated using an isotope pattern simulator (JEOL, Isotope Pattern Simulator).

Matrix assisted laser desorption/ionization (MALDI)-MS was performed using a spiral flight MS system (JEOL, JMS-S3000) with a semiconductor laser (wavelength: 349 nm) as the light source. DCTB[7] was used for the matrix.

Ultraviolet-visible (UV-vis) optical absorption spectra were measured using a PDA detector (Shimadzu, SPD-20A) attached to the chromatograph or a double-beam spectrometer (JASCO, V-630). Toluene or dichloromethane was used as solvent.

Inductively coupled plasma (ICP)-MS was performed with a spectrometer (Agilent Technologies, Agilent 7500c). Au, Pd and Bi were used as the internal standard.

X-ray photoelectron spectroscopy (XPS) spectra were collected using an electron spectrometer (JEOL, JPS-9010MC) at a base pressure of ~2×10^{-8} Torr. X-rays from the Mg-Kα line (1253.6 eV) were used for excitation. Silver plate was used as a substrate. The spectra were calibrated with the peak energies of Ag $3d_{5/2}$ (367.9 eV).

The Au L_3-edge X-ray absorption fine structure (XAFS) measurements were performed at beamlines BL01B1 of the SPring-8 facility of the Japan Synchrotron Radiation Research Institute (Proposal numbers 2021B1163, 2021A1102 and 2020A0695). The incident X-ray beam was monochromatized by a Si(111) double-crystal monochromator. The XAFS spectra of Au foil (Au L_3-edge) as reference was recorded in transmission mode using ionization chambers at room temperature. The Au L_3-edge XAFS spectra of the $[Au_{26}Pd(SPh^tBu)_{20}]^0$ and $[Au_{28}(SPh^tBu)_{20}]^0$ were measured in transmission mode using a 19-element Ge solid-state detector at 10 K. The X-ray energies for the Au L_3 edges were calibrated using Au foil. The X-ray absorption near edge structure (XANES) and extended X-ray absorption fine structure (EXAFS) spectra were analyzed using the xTunes program (version 2.5.9, Rigaku) as follows. The χ spectra were extracted by subtracting the atomic absorption background by cubic spline interpolation and normalized to the edge height. The normalized data were used as the XANES spectra. The k^3 -weighted χ spectra in the k range 3.0−14.0 Å^{-1} was Fourier-transformed (FT) into r space for structural analysis.

S1.6. Analysis

The average numbers of the exchanged ligands, x_{ave}, were estimated as

$$x_{\mathrm{ave}} = \sum_{x=0}^{18} x \frac{I(x)}{\left(\sum_{y=0}^{18} I(y)\right)}$$

Where x and y are the number of exchanged ligands and $I(x)$ and $I(x)$ are the areas observed in the RP-HPLC chromatogram (Figure 4, 8, and 9). The maximum value of x and y are 14, 16, and 18 for the reaction starting from $[Au_{25}(SC_2H_4Ph)_{18}]^-$ (Figure 4), $[Au_{24}Pd(SC_2H_4Ph)_{18}]^0$ (Figure 8), and $[Au_{24}Pt(SC_2H_4Ph)_{18}]^0$ (Figure 9), respectively.

S2. Additional Tables

Table S1. Retention Times of Each Metal NC Produced by Reaction between $[Au_{25}(SC_2H_4Ph)_{18}]^-$ and ^{t}BuPhSH.

Retention time (min) [a]	Chemical composition [b]
16.0	$[Au_{25}(SC_2H_4Ph)_{18}]^-$
34.1	$[Au_{25}(SC_2H_4Ph)_{18}]^0$
35.9	$[Au_{25}(SC_2H_4Ph)_{17}(SPh^tBu)_1]^0$
37.4	$[Au_{25}(SC_2H_4Ph)_{16}(SPh^tBu)_2]^0$
39.0	$[Au_{25}(SC_2H_4Ph)_{15}(SPh^tBu)_3]^0$
40.4	$[Au_{25}(SC_2H_4Ph)_{14}(SPh^tBu)_4]^0$
42.1	$[Au_{25}(SC_2H_4Ph)_{13}(SPh^tBu)_5]^0$
43.4	$[Au_{25}(SC_2H_4Ph)_{12}(SPh^tBu)_6]^0$
44.8	$[Au_{25}(SC_2H_4Ph)_{11}(SPh^tBu)_7]^0$
46.0	$[Au_{25}(SC_2H_4Ph)_{10}(SPh^tBu)_8]^0$
47.3	$[Au_{25}(SC_2H_4Ph)_9(SPh^tBu)_9]^0$
48.3	$[Au_{25}(SC_2H_4Ph)_8(SPh^tBu)_{10}]^0$
49.5	$[Au_{25}(SC_2H_4Ph)_7(SPh^tBu)_{11}]^0$
50.6	$[Au_{25}(SC_2H_4Ph)_6(SPh^tBu)_{12}]^0$
51.3	$[Au_{25}(SC_2H_4Ph)_5(SPh^tBu)_{13}]^0$
52.3	$[Au_{25}(SC_2H_4Ph)_4(SPh^tBu)_{14}]^0$
55.6	$[Au_{23}(SPh^tBu)_{17}]^0$
59.7	$[Au_{28}(SPh^tBu)_{20}]^0$

[a] See Figure 4. SPh^{t}Bu = 4-*tert*-butylbenzenethiolate (Scheme S1(b)). [b] Products exchanged with large number of the ligands were not necessarily produced in the reaction at short time (see Figure 4).

Table S2. Retention Times of Each Metal NC Produced by Reaction between $[Au_{24}Pd(SC_2H_4Ph)_{18}]^0$ and ^{t}BuPhSH.

Retention time (min) [a]	Chemical composition [b]
34.7	$[Au_{24}Pd(SC_2H_4Ph)_{18}]^0$
36.5	$[Au_{24}Pd(SC_2H_4Ph)_{17}(SPh^tBu)_1]^0$
37.7	$[Au_{24}Pd(SC_2H_4Ph)_{16}(SPh^tBu)_2]^0$
39.2	$[Au_{24}Pd(SC_2H_4Ph)_{15}(SPh^tBu)_3]^0$
40.6	$[Au_{24}Pd(SC_2H_4Ph)_{14}(SPh^tBu)_4]^0$
41.9	$[Au_{24}Pd(SC_2H_4Ph)_{13}(SPh^tBu)_5]^0$
43.4	$[Au_{24}Pd(SC_2H_4Ph)_{12}(SPh^tBu)_6]^0$
44.9	$[Au_{24}Pd(SC_2H_4Ph)_{11}(SPh^tBu)_7]^0$
46.0	$[Au_{24}Pd(SC_2H_4Ph)_{10}(SPh^tBu)_8]^0$
47.4	$[Au_{24}Pd(SC_2H_4Ph)_9(SPh^tBu)_9]^0$
47.4	$[Au_{24}Pd(SC_2H_4Ph)_8(SPh^tBu)_{10}]^0$
49.5	$[Au_{24}Pd(SC_2H_4Ph)_7(SPh^tBu)_{11}]^0$
50.3	$[Au_{24}Pd(SC_2H_4Ph)_6(SPh^tBu)_{12}]^0$
51.5	$[Au_{24}Pd(SC_2H_4Ph)_5(SPh^tBu)_{13}]^0$
52.8	$[Au_{24}Pd(SC_2H_4Ph)_4(SPh^tBu)_{14}]^0$
54.1	$[Au_{24}Pd(SC_2H_4Ph)_3(SPh^tBu)_{15}]^0$

55.3	$[Au_{24}Pd(SC_2H_4Ph)_2(SPh^tBu)_{16}]^0$
57.4	$[Au_{26}Pd(SPh^tBu)_{20}]^0$

[a] See Figure 8. SPh^tBu = 4-*tert*-butylbenzenethiolate. [b] Products exchanged with large number of the ligands were not necessarily produced in the reaction at short time (see Figure 8).

Table S3. Retention Times of Each Metal NC Produced by Reaction between $[Au_{24}Pt(SC_2H_4Ph)_{18}]^0$ and ^tBuPhSH.

Retention time (min) [a]	Chemical composition [b]
34.4	$[Au_{24}Pt(SC_2H_4Ph)_{18}]^0$
35.9	$[Au_{24}Pt(SC_2H_4Ph)_{17}(SPh^tBu)_1]^0$
37.7	$[Au_{24}Pt(SC_2H_4Ph)_{16}(SPh^tBu)_2]^0$
39.0	$[Au_{24}Pt(SC_2H_4Ph)_{15}(SPh^tBu)_3]^0$
40.7	$[Au_{24}Pt(SC_2H_4Ph)_{14}(SPh^tBu)_4]^0$
42.3	$[Au_{24}Pt(SC_2H_4Ph)_{13}(SPh^tBu)_5]^0$
43.5	$[Au_{24}Pt(SC_2H_4Ph)_{12}(SPh^tBu)_6]^0$
44.7	$[Au_{24}Pt(SC_2H_4Ph)_{11}(SPh^tBu)_7]^0$
46.1	$[Au_{24}Pt(SC_2H_4Ph)_{10}(SPh^tBu)_8]^0$
47.5	$[Au_{24}Pt(SC_2H_4Ph)_9(SPh^tBu)_9]^0$
48.9	$[Au_{24}Pt(SC_2H_4Ph)_8(SPh^tBu)_{10}]^0$
49.8	$[Au_{24}Pt(SC_2H_4Ph)_7(SPh^tBu)_{11}]^0$
50.5	$[Au_{24}Pt(SC_2H_4Ph)_6(SPh^tBu)_{12}]^0$
51.4	$[Au_{24}Pt(SC_2H_4Ph)_5(SPh^tBu)_{13}]^0$
52.5	$[Au_{24}Pt(SC_2H_4Ph)_4(SPh^tBu)_{14}]^0$
53.3	$[Au_{24}Pt(SC_2H_4Ph)_3(SPh^tBu)_{15}]^0$
54.1	$[Au_{24}Pt(SC_2H_4Ph)_2(SPh^tBu)_{16}]^0$
54.9	$[Au_{24}Pt(SC_2H_4Ph)_1(SPh^tBu)_{17}]^0$
55.6	$[Au_{24}Pt(SPh^tBu)_{18}]^0$

[a] See Figure 9. SPh^tBu = 4-*tert*-butylbenzenethiolate. [b] Products exchanged with large number of the ligands were not necessarily produced in the reaction at short time (see Figure 9).

S3. Additional Schemes

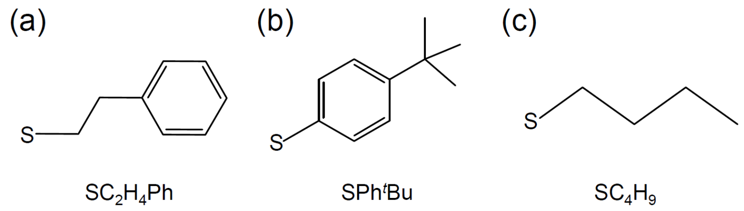

Scheme S1. Structures of (a) SC_2H_4Ph, (b) SPh^tBu and (c) SC_4H_9.

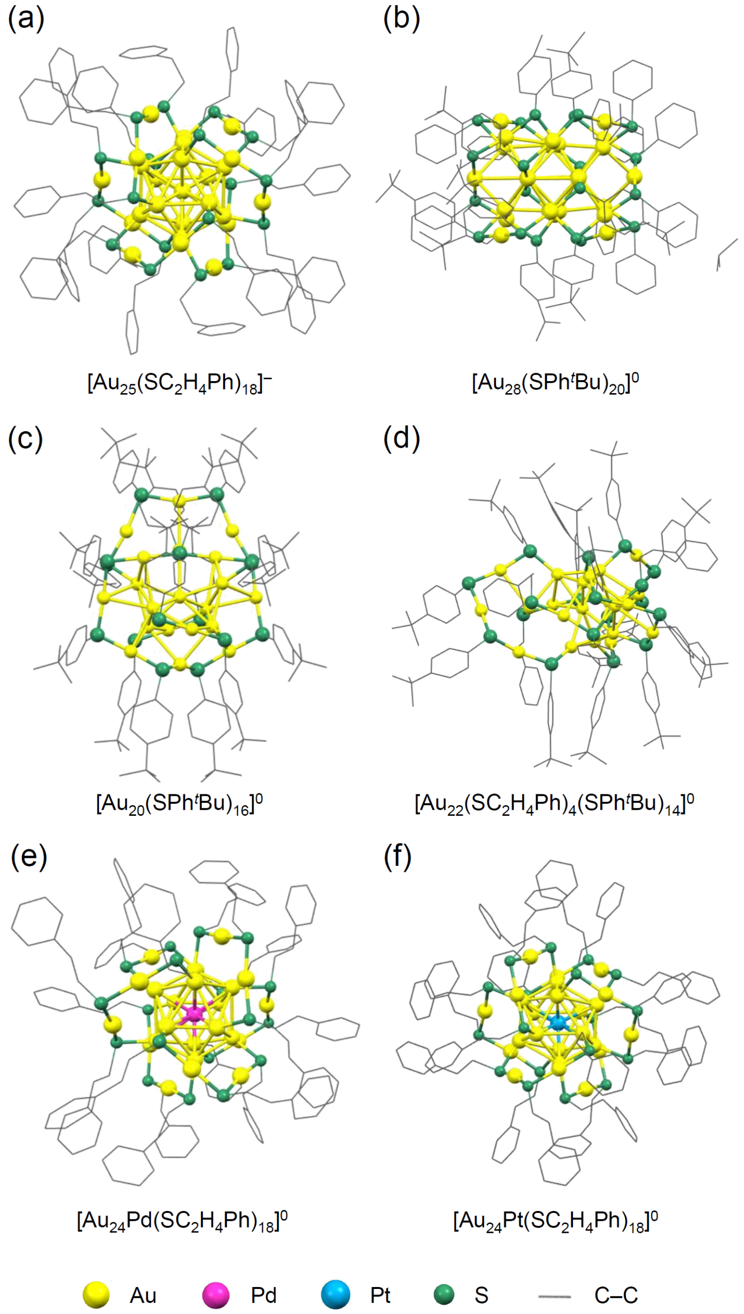

Scheme S2. Geometrical structures of (a) $[Au_{25}(SC_2H_4Ph)_{18}]^-$,[8] (b) $[Au_{28}(SPh^tBu)_{20}]^0$,[9] (c) $[Au_{20}(SPh^tBu)_{16}]^0$,[10] (d) $[Au_{22}(SC_2H_4Ph)_4(SPh^tBu)_{14}]^0$,[11] (e) $[Au_{24}Pd(SC_2H_4Ph)_{18}]^0$,[12] and (f) $[Au_{24}Pt(SC_2H_4Ph)_{18}]^0$.[12] Geometrical structures of (a)−(c), (e), and (f) were determined by single crystal X-ray diffraction, whereas that of (d) was estimated by density functional theory calculation. Reproduced from Refs. 8−12.

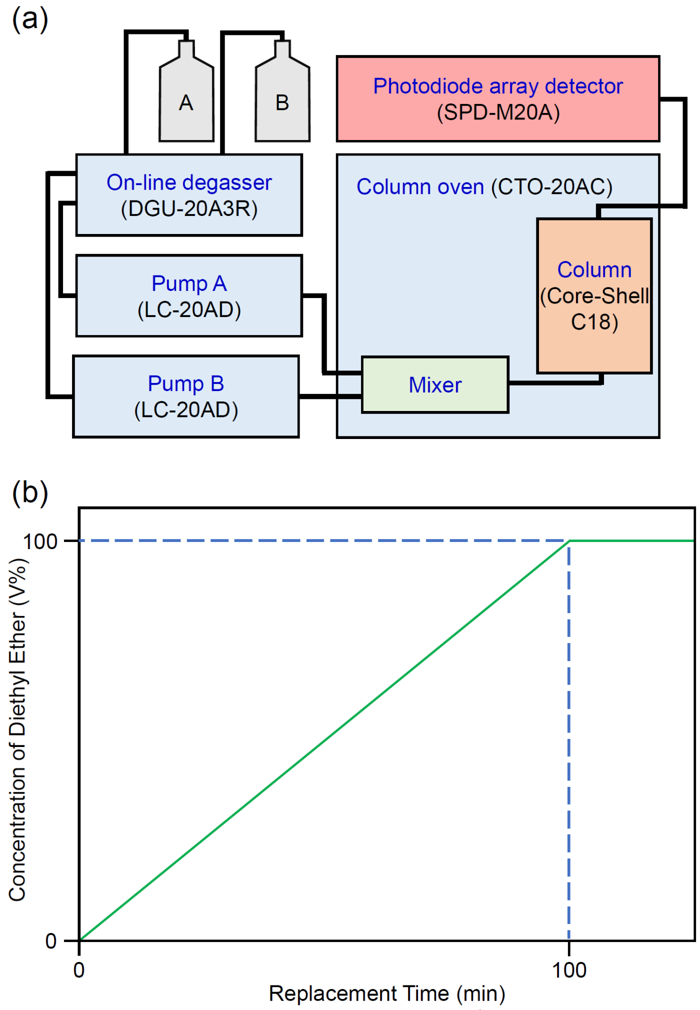

Scheme S3. (a) Schematic of HPLC system used in this work.[4] (b) Gradient program used for substitution of the mobile phase. The mobile phase was generally substituted from 100 V% of the adsorption solvent (acetonitrile) to 100 V% of the elution solvent (diethyl ether) within 100 min.

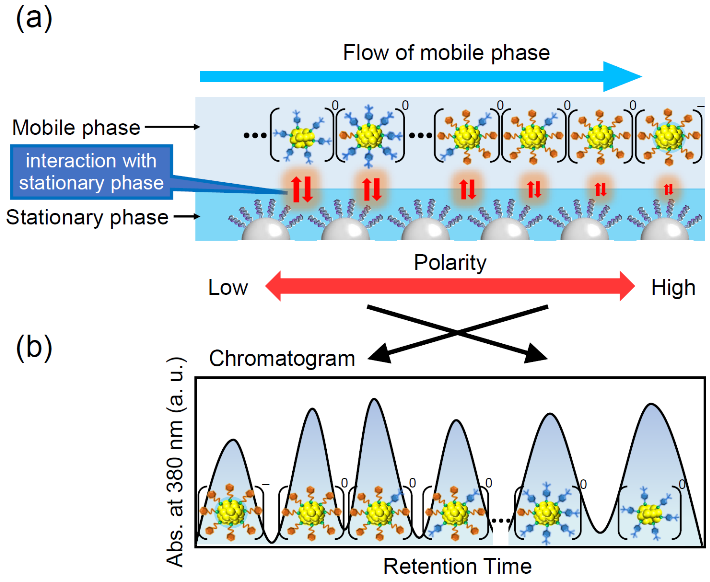

Scheme S4. Mechanism of the separation of $[Au_{25}(SC_2H_4Ph)_{18}]^-$, $[Au_{25}(SC_2H_4Ph)_{18-x}(SPh^tBu)_x]^0$, and $[Au_{23}(SPh^tBu)_{17}]^0$ by RP-HPLC; (a) the flow order of the metal NCs in RP-column and (b) the elution order of each metal NC in chromatogram.

S4. Additional Figures

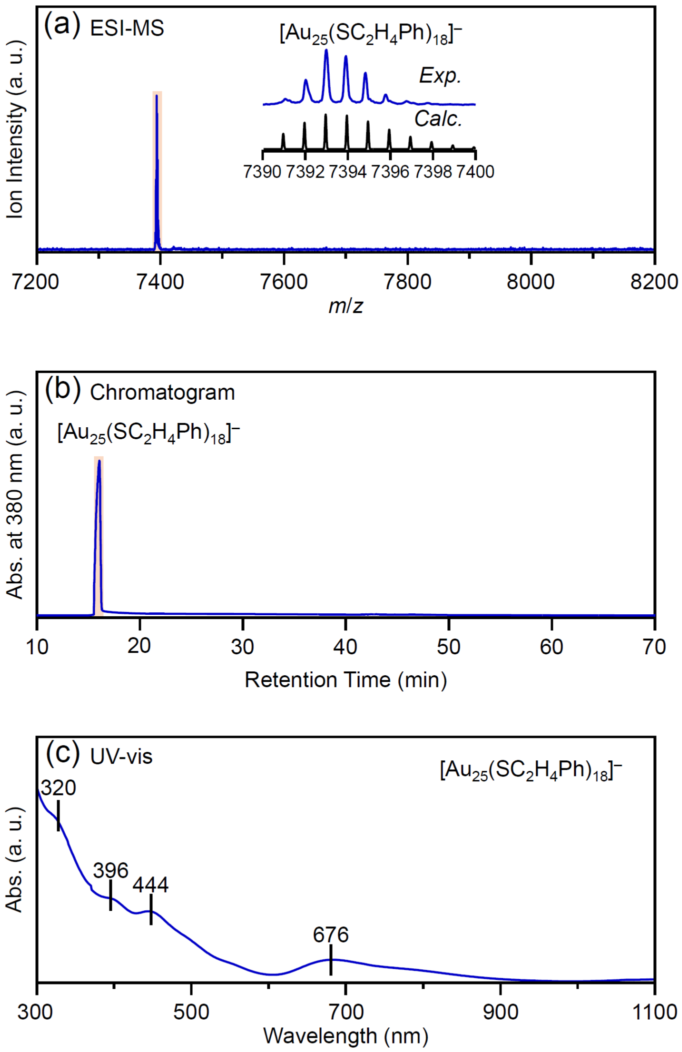

Figure S1. (a) Negative-ion ESI mass spectrum, (b) RP-HPLC chromatogram, and (c) UV-vis optical absorption spectrum of a precursor $[Au_{25}(SC_2H_4Ph)_{18}]^-$. In (a), the inset shows the comparison of the isotope pattern between experimental and simulated spectra.

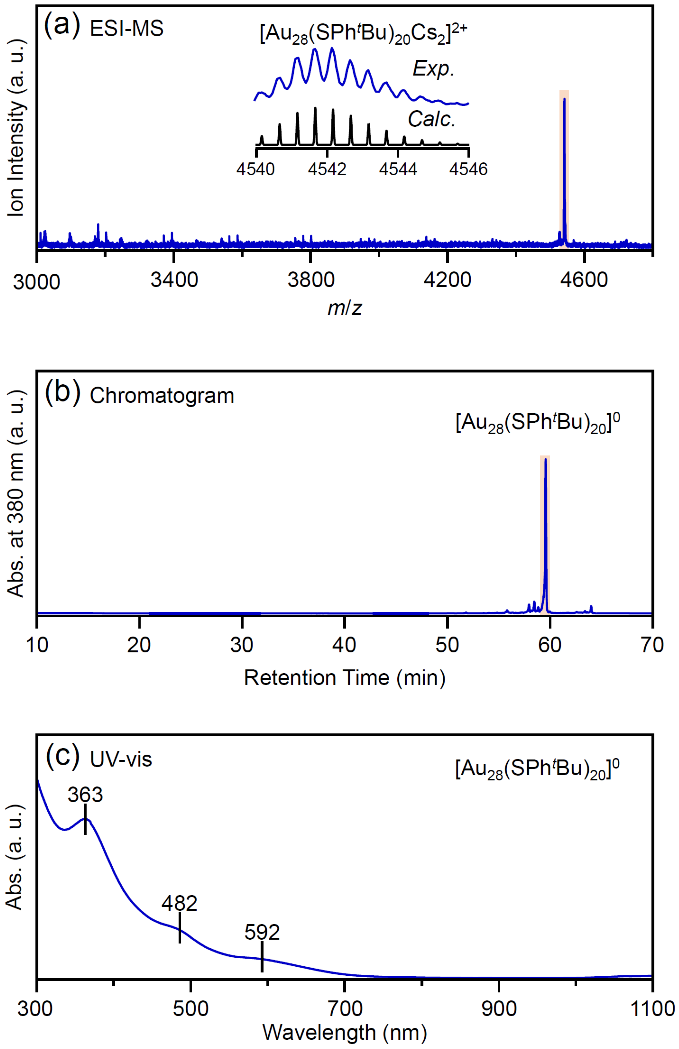

Figure S2. (a) Positive-ion ESI mass spectrum, (b) RP-HPLC chromatogram, and (c) UV-vis optical absorption spectrum of $[Au_{28}(SPh^tBu)_{20}]^0$, which was obtained by the reaction condition in Ref. 9 (Figure S10). In (a), the inset shows the comparison of the isotope pattern between the experimental and simulated spectra of $[Au_{28}(SPh^tBu)_{20}]^0(Cs^+)_2$.

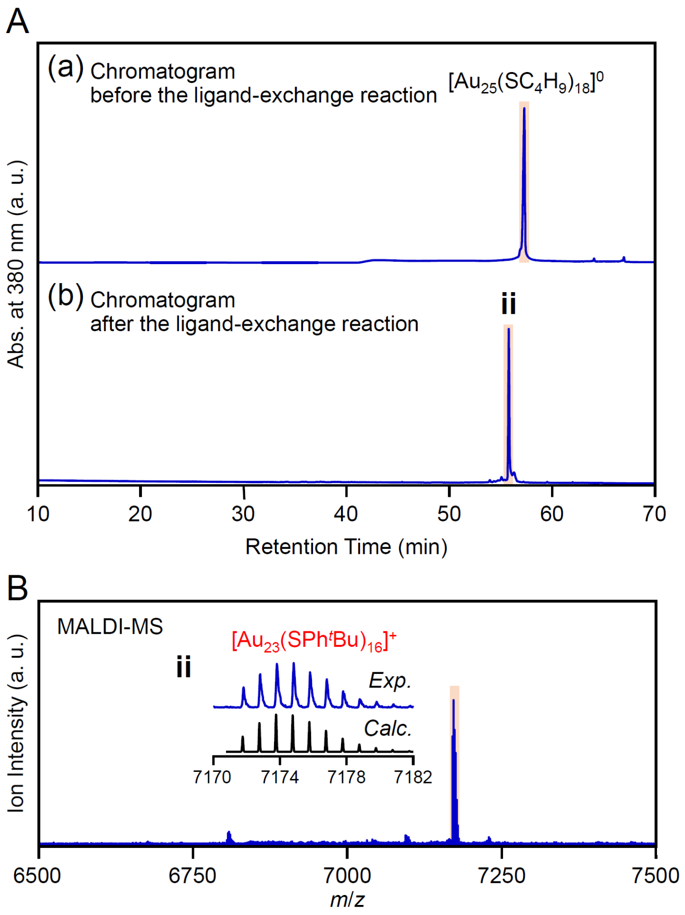

Figure S3. (A) RP-HPLC chromatogram of (a) before and (b) after the reaction between $[Au_{25}(SC_4H_9)_{18}]^0$ and ^{t}BuPhSH (**ii**). Note that the chromatogram (b) is not that for the as-prepared sample but for the isolated sample. (B) Positive-ion MALDI mass spectrum of the main products after the reaction ($[Au_{23}(SPh^tBu)_{17}]^0$). The inset shows the comparison of the isotope pattern between the experimental and simulated spectra. In (A)(a), the peak of $[Au_{25}(SC_4H_9)_{18}]^0$ (57.3 min) appears at longer retention time than that of $[Au_{25}(SC_2H_4Ph)_{18}]^-$ (14.7 min; Figure S1) because 1) the polarity of SC_4H_9 is lower than that of SC_2H_4Ph,[13] and 2) the neutral NC was used in this experiment (e. g. Figure 4(a)(f)).

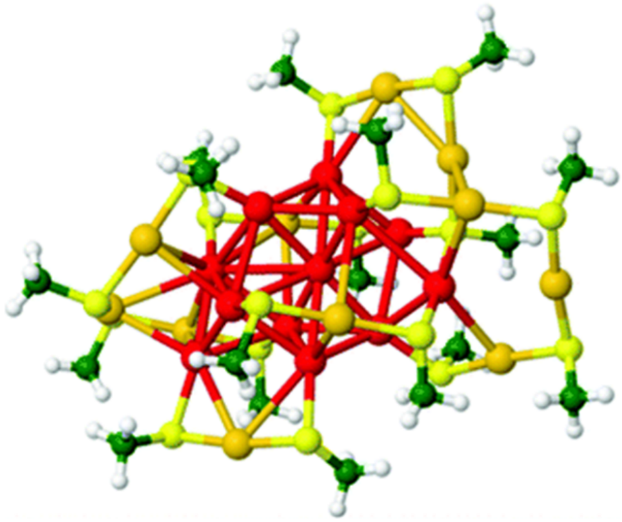

Figure S4. Reported, calculated geometrical structure on $[Au_{23}(SCH_3)_{17}]^0$ (SCH_3 = methanethiolate).[14] Au; red and orange, S; yellow, C; green, H; white. A different geometrical structure has also been reported for $[Au_{23}(SCH_3)_{17}]^0$ in Ref. 15. These geometrical structures might be a candidate structure for $[Au_{23}(SPh^tBu)_{17}]^0$. However, the geometrical structures of $Au_n(SR)_m$ NCs often varies depending on the functional-group structure of the thiolate.[16] Thus, it is indispensable to calculate the geometrical structure using SPh^tBu as a thiolate for more precisely estimating the geometrical structure of $[Au_{23}(SPh^tBu)_{17}]^0$. Reproduced with permission from Ref. 14. Copyright 2018 Royal Society of Chemistry.

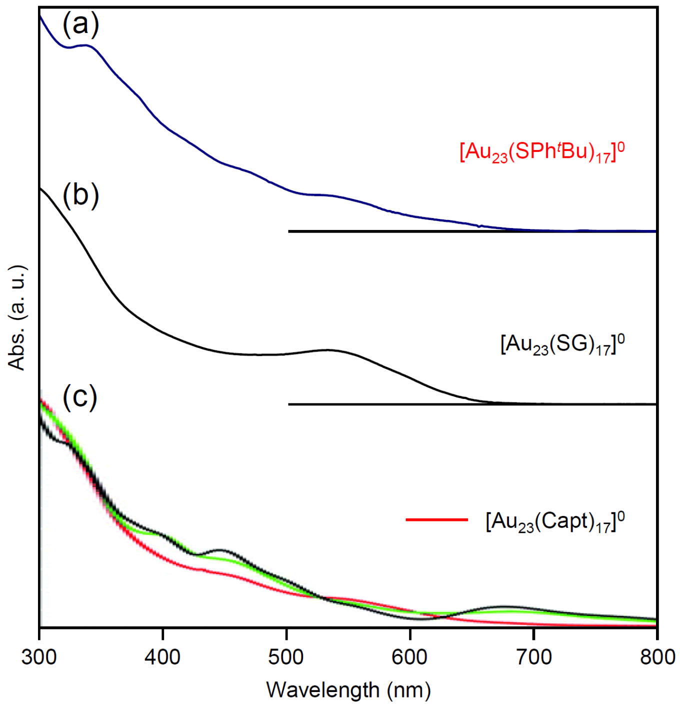

Figure S5. Comparison of UV-vis spectra between (a) $[Au_{23}(SPh^tBu)_{17}]^0$ (this work), (b) $[Au_{23}(SG)_{17}]^0$ (SG = glutathionate),[17] and (c) $[Au_{23}(Capt)_{17}]^0$ (Capt = captopril; red line)[14]. Although these spectra are overall similar each other, there are also a little difference in peaks structure depending on the functional-group structure of the thiolate. This result again indicates that it is indispensable to calculate the geometrical structure using SPh^tBu as a thiolate for precisely estimating the geometrical structure of $[Au_{23}(SPh^tBu)_{17}]^0$. Reproduced with permission from Ref. 14 and 17. Copyright 2018 Royal Society of Chemistry and Copyright 2018 Royal Society of Chemistry.

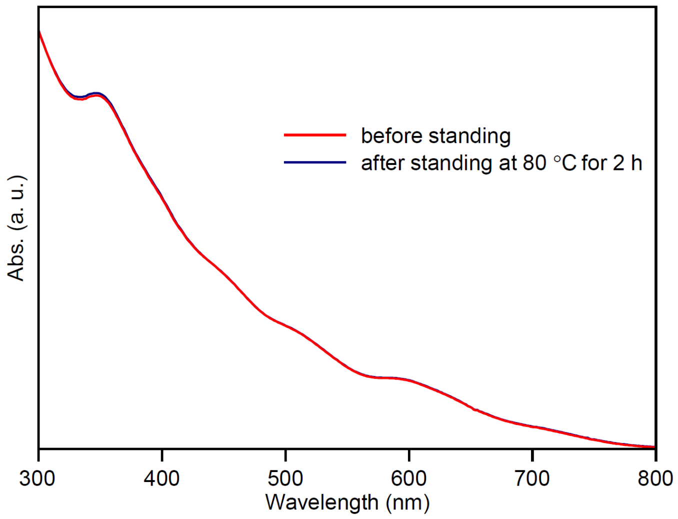

Figure S6. Comparison of UV-vis absorption spectra before and after standing the toluene solution of $[Au_{23}(SPh^{t}Bu)_{17}]^{0}$ at 80 °C for 2 h.

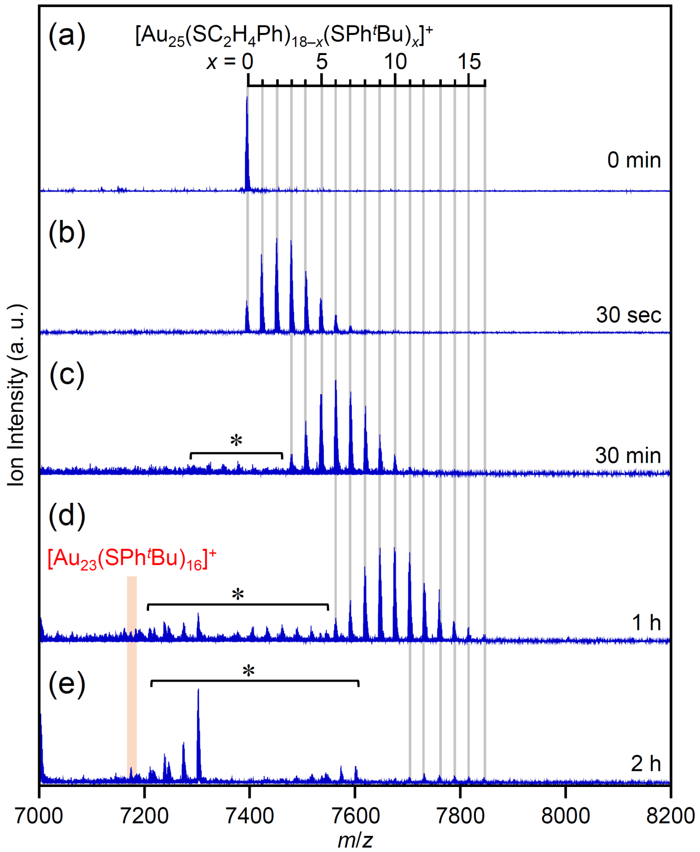

Figure S7. Positive-ion MALDI mass spectra of the products obtained by the reaction between $[Au_{25}(SC_2H_4Ph)_{18}]^-$ and ^{t}BuPhSH: (a) 0 min, (b) 30 sec, (c) 30 min, (d) 1 h, and (e) 2 h. In these spectra, the peaks with "*" are attributed to the fragments of the products caused by laser. A series of these MALDI mass spectra indicate that 1) the ligand-exchange reaction occurred in this experimental condition and that 2) the maximum number of the ligand exchange while keeping the number of Au atoms (25 atoms) is around 14. The peak originated to $[Au_{23}(SPh^tBu)_{17}]^0$, which was observed in RP-HPLC chromatogram (Figure 4), was observed with the quite weak intensity in these MALDI mass spectra. On the basis of the previous studies,[11,18–20] $[Au_{25}(SC_2H_4Ph)_{18-x}(SPh^tBu)_x]^0$ can be ionized without fragmentations in the MALDI-MS,[11] whereas $[Au_{23}(SPh^tBu)_{17}]^0$ NCs could not be ionized without fragmentations ($Au_n(SPh^tBu)_m$ NCs are typically observed in the form of $Au_n(SPh^tBu)_{m-1}$ NCs)[18–20] in the MALDI-MS. Thus, $[Au_{25}(SC_2H_4Ph)_{18-x}(SPh^tBu)_x]^0$ should be more easily monitored than $[Au_{23}(SPh^tBu)_{17}]^0$. This seems to be the reason why the peak originated to $[Au_{23}(SPh^tBu)_{17}]^0$ was observed with the quite weak intensity in these MALDI mass spectra, different from the peaks originated to $[Au_{25}(SC_2H_4Ph)_{18-x}(SPh^tBu)_x]^0$. These results indicate that the combined use of MALDI-MS and RP-HPLC is very important to elucidate the details of the ligand-exchange mechanism while estimating the product distribution for this reaction system.

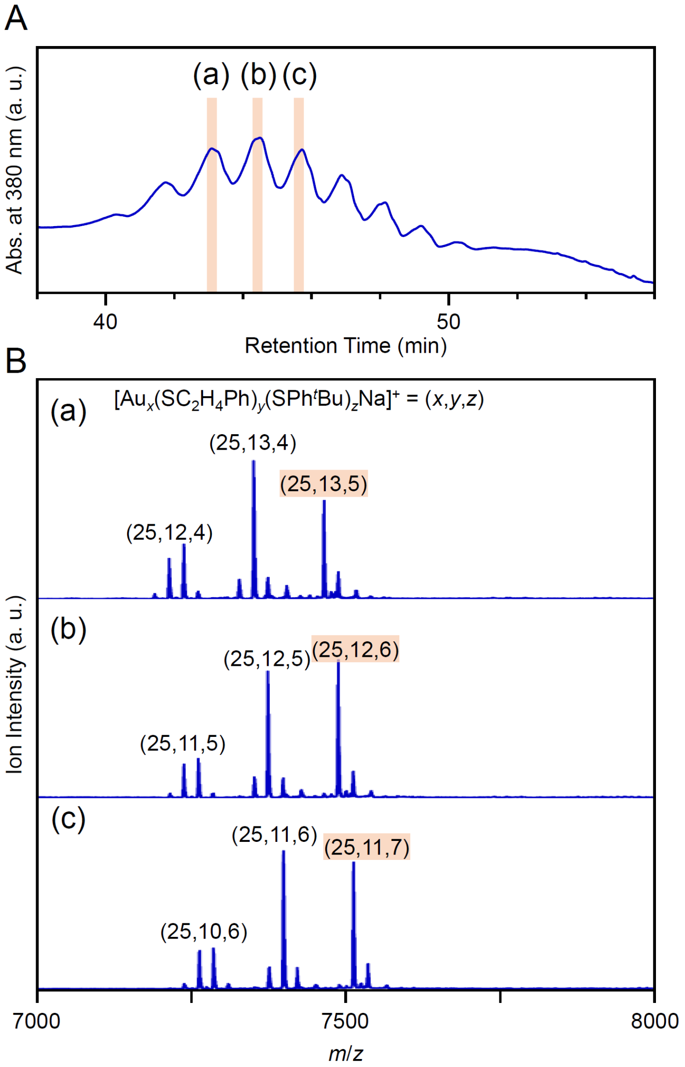

Figure S8. (A) RP-HPLC chromatogram of the reaction intermediates, $[Au_{25}(SC_2H_4Ph)_{18-x}(SPh^tBu)_x]^0$ (x = 4−7). (B) Positive-ion ESI mass spectrum of each fraction of (a)−(c) in RP-HPLC chromatogram (A). These mass spectra were obtained by LC/MS, in which ESI mass spectrometer was directly connected to HPLC apparatus.[4,17] Each ESI mass spectrum in B(a)−(c) includes the fragment ions produced in ESI process (ligand desorption), in addition to the peaks originated to mother $[Au_{25}(SC_2H_4Ph)_{18-x}(SPh^tBu)_x]^0$ (highlighted with red marks), indicating that monitoring the mass distribution of the intermediary $[Au_{25}(SC_2H_4Ph)_{18-x}(SPh^tBu)_x]^0$ NCs is difficult even using ESI-MS.

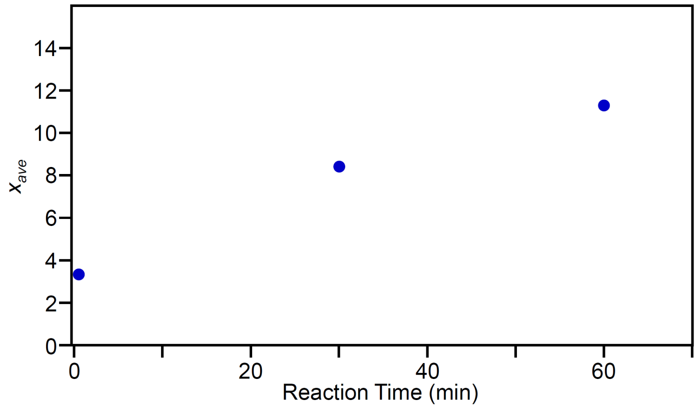

Figure S9. Plots of average number of exchanged ligands, x_{ave}, against reaction time for the reaction between $[Au_{25}(SC_2H_4Ph)_{18}]^0$ and ^{t}BuPhSH (see S1.6. Analysis). The x_{ave} does not linearly increase with the reaction time, indicating that this reaction cannot be described by the pseudo-first-order reaction. Probably, the reverse reaction also occurred in parallel.

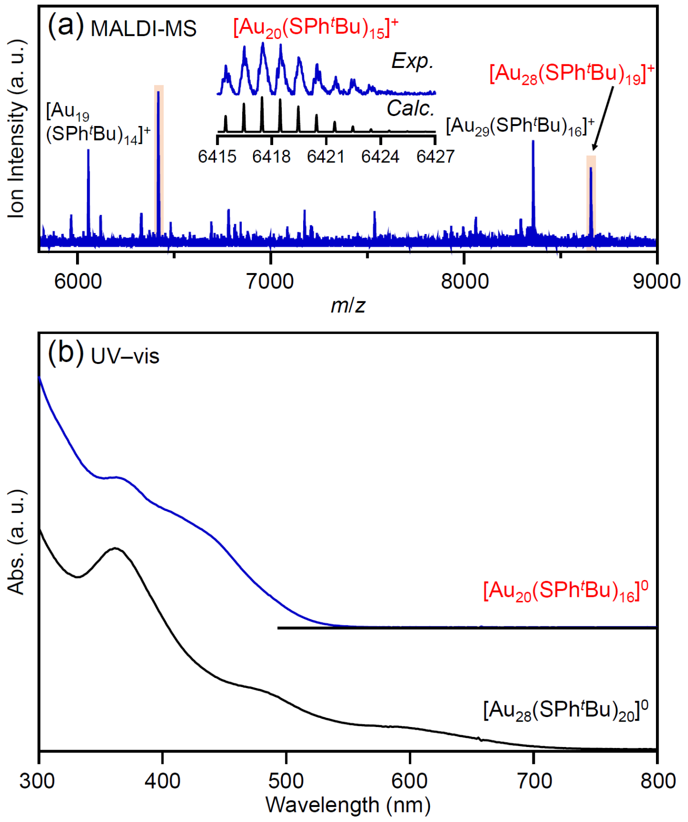

Figure S10. (a) Positive-ion MALDI mass spectrum of the sample shown in Figure 3(c). The inset shows the comparison of the isotope pattern between the experimental and simulated spectra. This figure demonstrates that the peak at 51.6 and 59.7 min can be assigned to $[Au_{20}(SPh^tBu)_{16}]^0$ and $[Au_{28}(SPh^tBu)_{20}]^0$, respectively, whose synthesis has been reported by Jin group[9,10]. Note that $[Au_{20}(SPh^tBu)_{16}]^0$ and $[Au_{28}(SPh^tBu)_{20}]^0$ were observed as $[Au_{20}(SPh^tBu)_{15}]^+$ and $[Au_{28}(SPh^tBu)_{19}]^+$, respectively, in the MALDI mass spectrum since $[Au_n(SPh^tBu)_m]^0$ NCs typically cause the release of one SPh^{t}Bu ligand when irradiated with laser.[18–20] (b) Optical absorption spectrum obtained using PDA detector for $[Au_{20}(SPh^tBu)_{16}]^0$ and $[Au_{28}(SPh^tBu)_{20}]^0$. To best of our knowledge, this is a first report on the optical absorption spectrum of $[Au_{20}(SPh^tBu)_{16}]^0$.

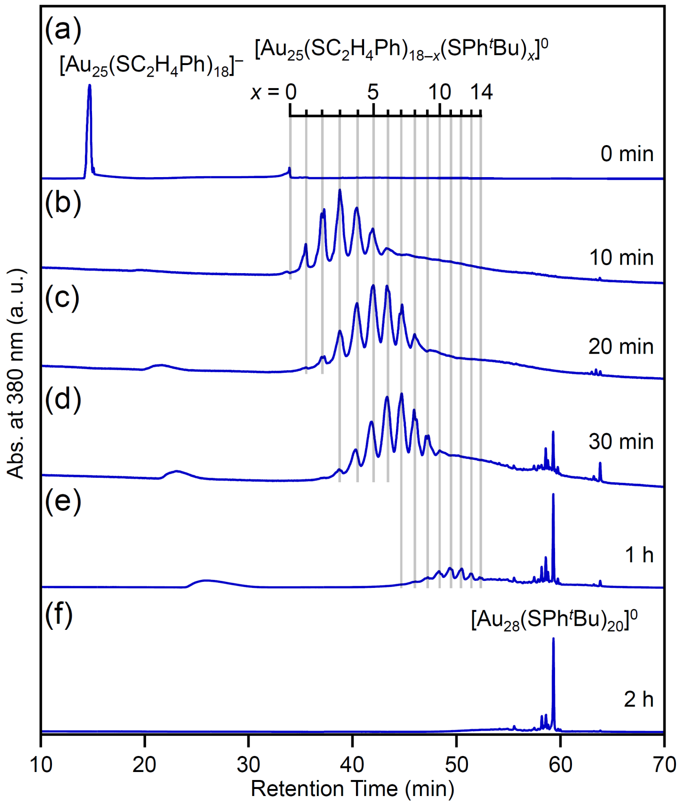

Figure S11. RP-HPLC chromatogram of the products obtained by the reaction between $[Au_{25}(SC_2H_4Ph)_{18}]^-$ and ^{t}BuPhSH ([^{t}BuPhSH]/[SC_2H_4Ph] ≅ 250) at 80 °C (similar experimental condition as that in Ref. 9; Scheme 1(a)): (a) 0 min, (b) 10 min, (c) 20 min, (d) 30 min, (e) 1 h, and (f) 2 h. The ligand-exchange products eluted at longer retention times than $[Au_{25}(SC_2H_4Ph)_{18}]^0$ because the polarity of SPh^{t}Bu is lower than that of SC_2H_4Ph.[13] The fine peak progression in each major peak is caused by the existence of topological isomers.[21] A series of RP-HPLC chromatogram demonstrates that 1) $[Au_{25}(SC_2H_4Ph)_{18}]^-$ was first oxidized to the neutral $[Au_{25}(SC_2H_4Ph)_{18}]^0$, that 2) then the ligand exchange occurred, that 3) the maximum number of the ligand exchange while keeping the number of Au atoms (25 atoms) is 14, and that 4) then $[Au_{28}(SPh^tBu)_{20}]^0$ was produced. In this experimental condition, negligible quantity of $[Au_{23}(SPh^tBu)_{17}]^0$ (55.6 min) was produced.

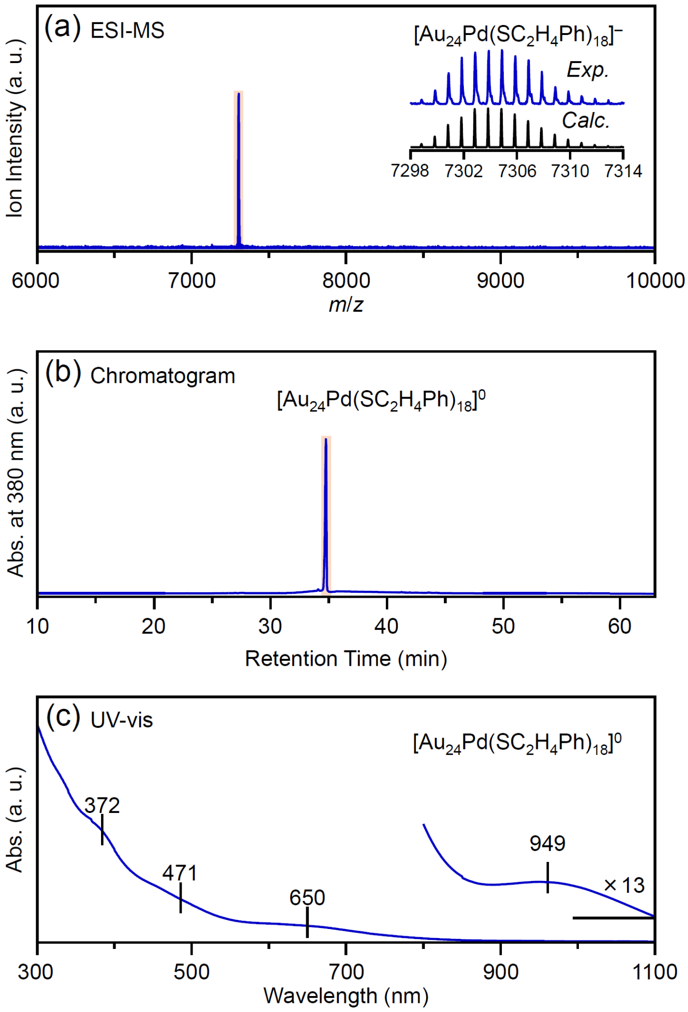

Figure S12. (a) Negative-ion ESI mass spectrum, (b) RP-HPLC chromatogram, and (c) UV-vis optical absorption spectrum of a precursor $[Au_{24}Pd(SC_2H_4Ph)_{18}]^0$. In (a), the inset shows the comparison of the isotope pattern between the experimental and simulated spectra. In this MS, Cs^+ ion was not added to the sample solution, leading to the observation of $[Au_{24}Pd(SC_2H_4Ph)_{18}]^0$ as a $[Au_{24}Pd(SC_2H_4Ph)_{18}]^-$ due to the ionization during ESI process.[22] In (b), $[Au_{24}Pd(SC_2H_4Ph)_{18}]^0$ eluted at the retention time of 34.7 min (Figure 6A(a)). This retention time is longer than that of $[Au_{25}(SC_2H_4Ph)_{18}]^-$ (Figure 1A(a)) since $[Au_{24}Pd(SC_2H_4Ph)_{18}]^0$ is neutral NC, whereas $[Au_{25}(SC_2H_4Ph)_{18}]^-$ is anion NC (e. g. Figure 4(a)(f)).

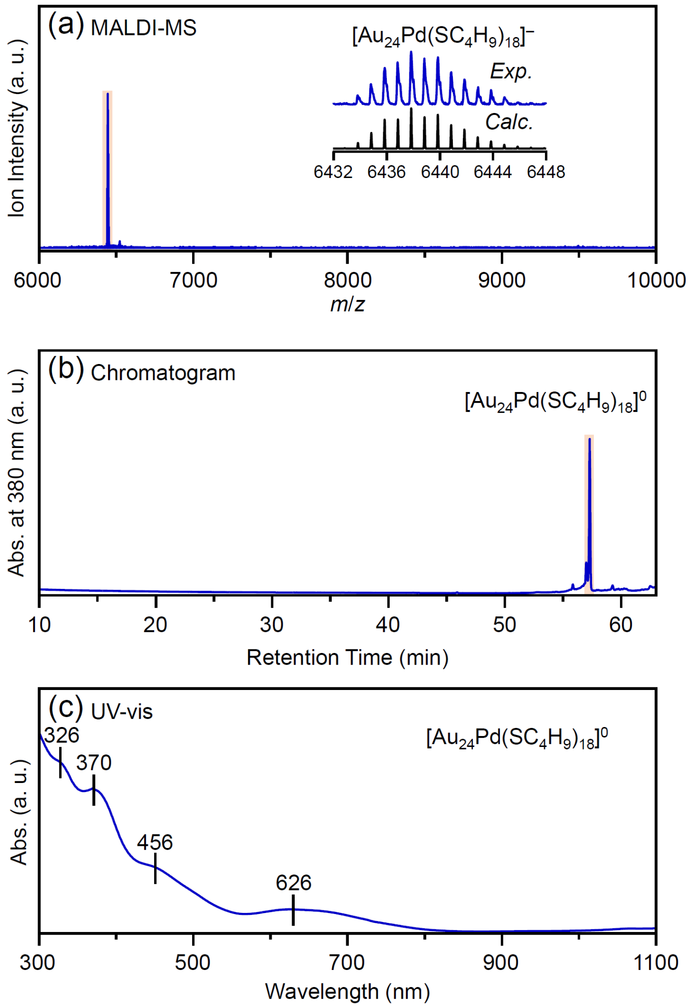

Figure S13. (a) Negative-ion ESI mass spectrum, (b) RP-HPLC chromatogram, and (c) optical absorption spectrum of a precursor $[Au_{24}Pd(SC_4H_9)_{18}]^0$ (SC_4H_9 = 1-buthanethiolate). In (a), the inset shows the comparison of the isotope pattern between the experimental and simulated spectra. In this MS, Cs^+ ion was not added to the sample solution, leading to the observation of $[Au_{24}Pd(SC_4H_9)_{18}]^0$ as a $[Au_{24}Pd(SC_4H_9)_{18}]^-$ due to the ionization during ESI process.[22]

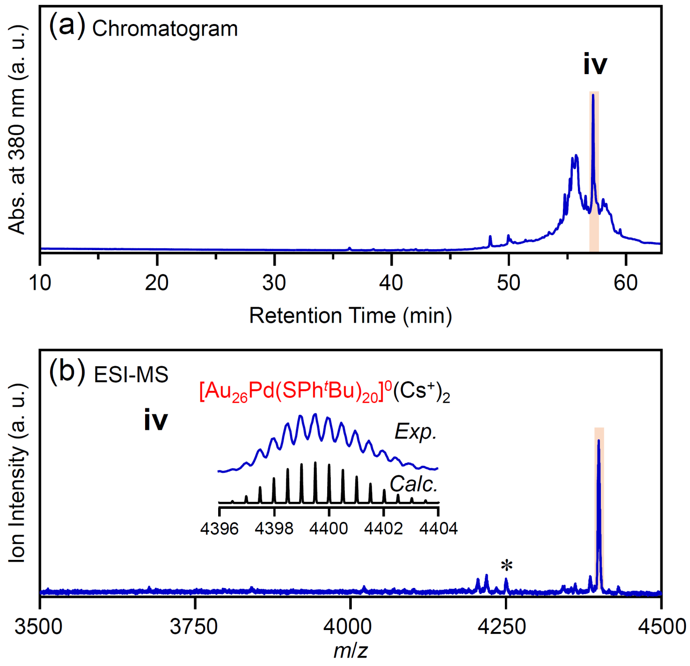

Figure S14. (a) RP-HPLC chromatogram and (b) positive-ion ESI mass spectrum of the products obtained by the reaction between $[Au_{24}Pd(SC_4H_9)_{18}]^0$ and ^{t}BuPhSH (**iv**). In the ESI-MS, Cs^+ ion was added as a cation source. The inset shows the comparison of the isotope pattern between the experimental and simulated spectra. The minor peak with (*) is assigned to $[Au_{27}Pd(SPh^tBu)_{17}]^0(Cs^+)_2$.

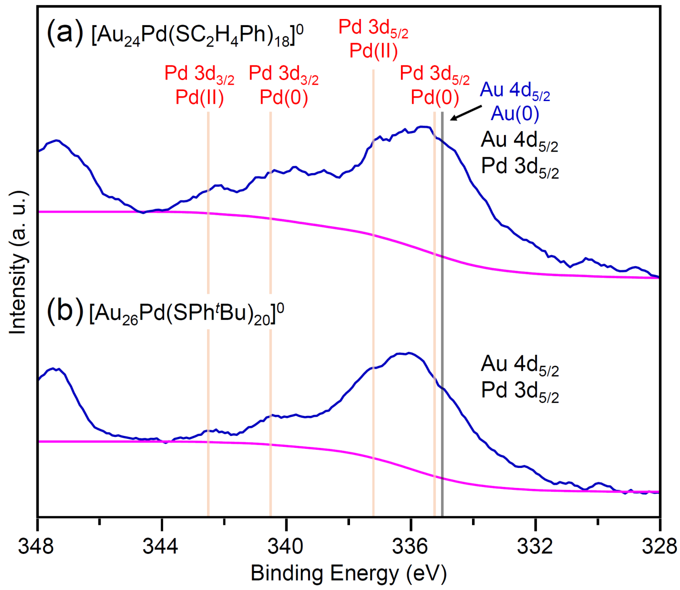

Figure S15. Pd 3d XPS spectra of (a) $[Au_{24}Pd(SC_2H_4Ph)_{18}]^0$ and (b) $[Au_{26}Pd(SPh^tBu)_{20}]^0$ (**iii**). This figure demonstrates that the ligand-exchange product ((b); $[Au_{26}Pd(SPh^tBu)_{20}]^0$) certainly includes Pd atom, similar to a precursor $[Au_{24}Pd(SC_2H_4Ph)_{18}]^0$ (a).

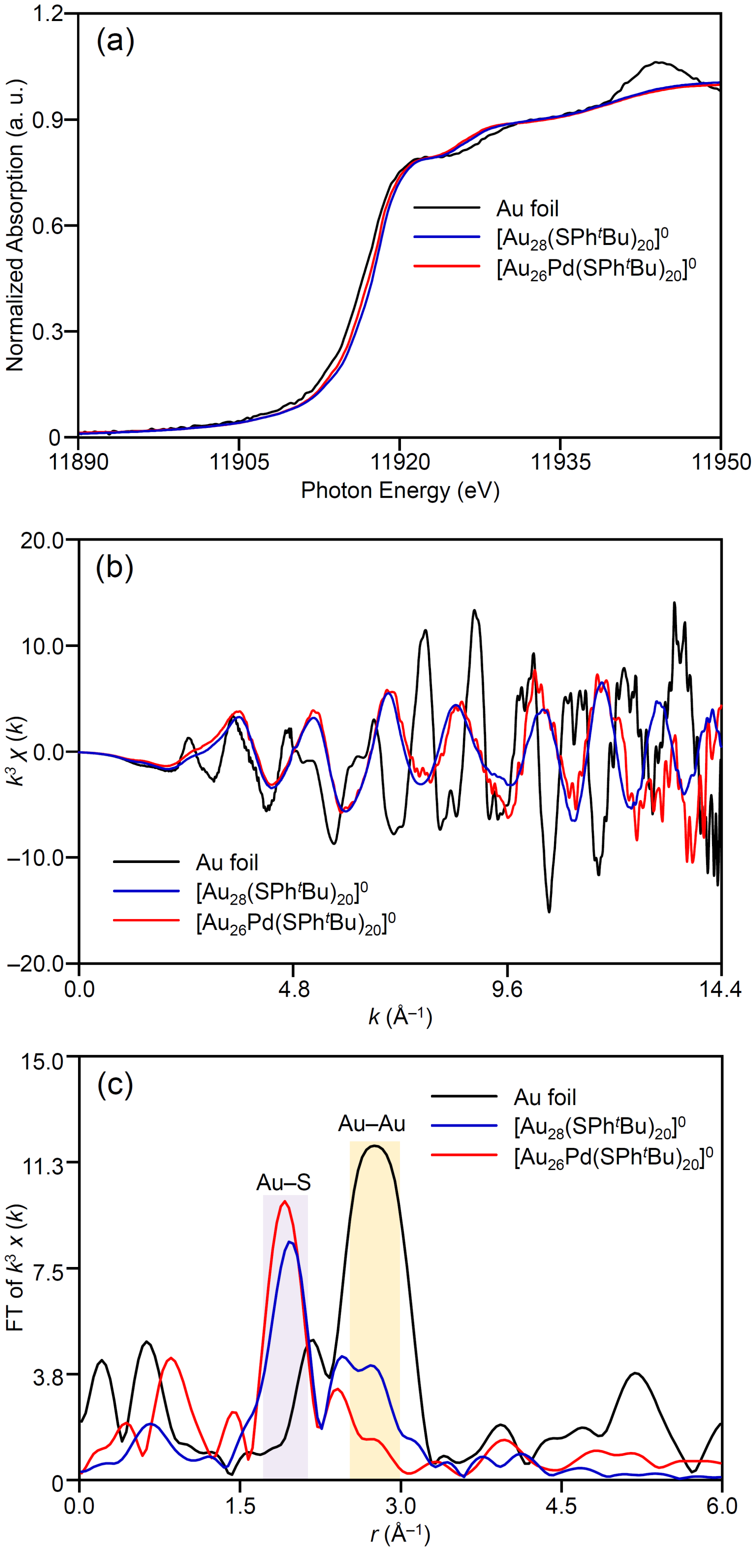

Figure S16. Results of Au L_3-edge XAFS analysis for $[Au_{26}Pd(SPh^tBu)_{20}]^0$ together with those for $[Au_{28}(SPh^tBu)_{20}]^0$ and Au foil: (a) XANES, (b) EXAFS, and (c) FT-EXAFS spectra. The XANES spectra demonstrate that Au in $[Au_{26}Pd(SPh^tBu)_{20}]^0$ is a little reduced than Au in mono-Au $[Au_{28}(SPh^tBu)_{20}]^0$ probably due to the charge transfer from Pd to Au. FT-EXAFS spectra demonstrate that both Au−S and Au−Au bonds also exist in $[Au_{26}Pd(SPh^tBu)_{20}]^0$ as well as in $[Au_{28}(SPh^tBu)_{20}]^0$. In this study, we have also attempted to obtain the information for Au−Pd bond by Pd-edge XAFS analysis. However, we could not obtain the reliable information on Au−Pd bond in $[Au_{26}Pd(SPh^tBu)_{20}]^0$ since the number of Pd in $[Au_{26}Pd(SPh^tBu)_{20}]^0$ is only one and thereby the S/N ratio in the spectrum was quite bad.

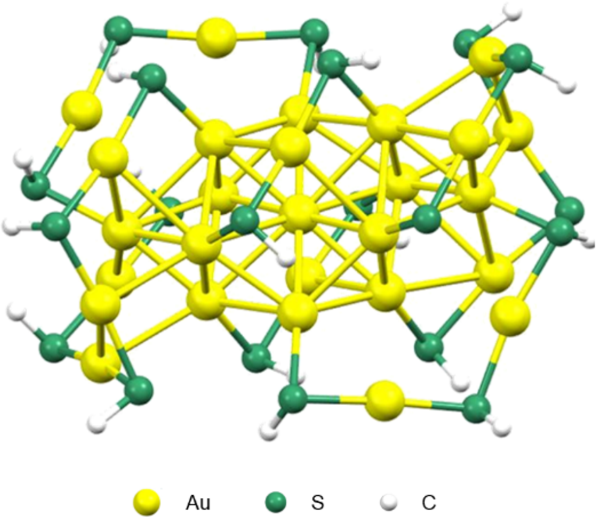

Figure S17. Reported, calculated geometrical structure for $[Au_{27}(SCH_3)_{20}]^-$.[23] This structure has one central Au atom. This Au atom might be replaced with Pd atom in $[Au_{26}Pd(SPh^tBu)_{20}]^0$. However, the geometrical structures of $Au_n(SR)_m$ NCs often varies depending on the functional-group structure of the thiolate.[16] Thus, it is indispensable to calculate the geometrical structure using SPh^tBu as a thiolate for precisely estimating the geometrical structure of $[Au_{26}Pd(SPh^tBu)_{20}]^0$.

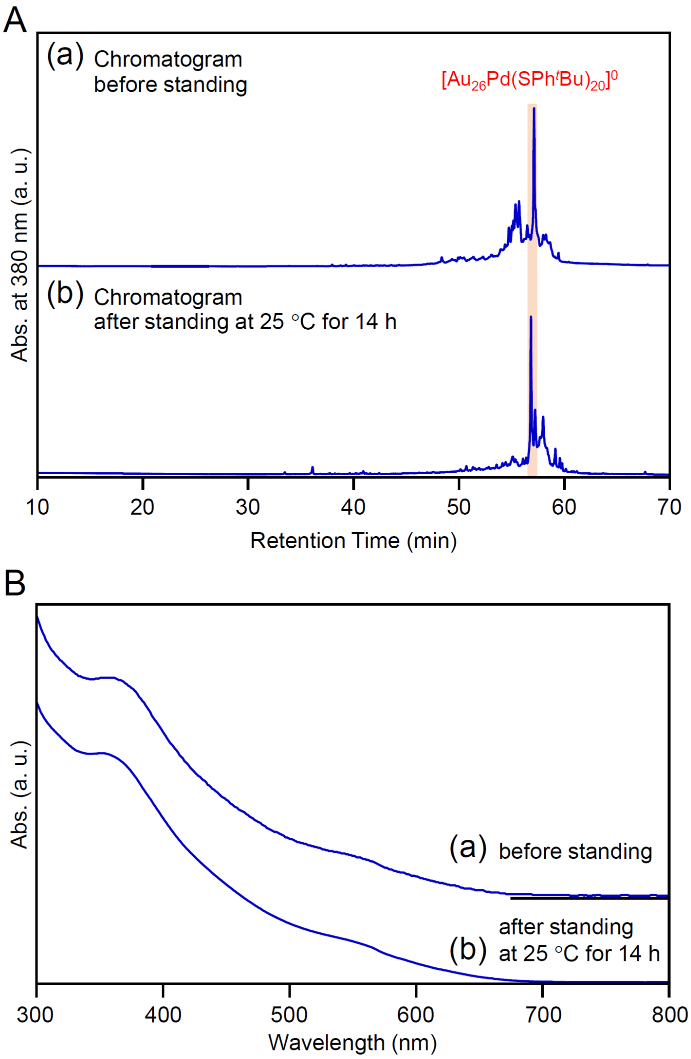

Figure S18. Stability test of $[Au_{26}Pd(SPh^tBu)_{20}]^0$ in toluene solution investigated by (A) RP-HPLC and (B) optical absorption spectroscopy; (a) before and (b) after leaving in the toluene solution at 25 °C for 14 h. These results indicate that $[Au_{26}Pd(SPh^tBu)_{20}]^0$ can keep the chemical composition in toluene solution at 25 °C for 14 h.

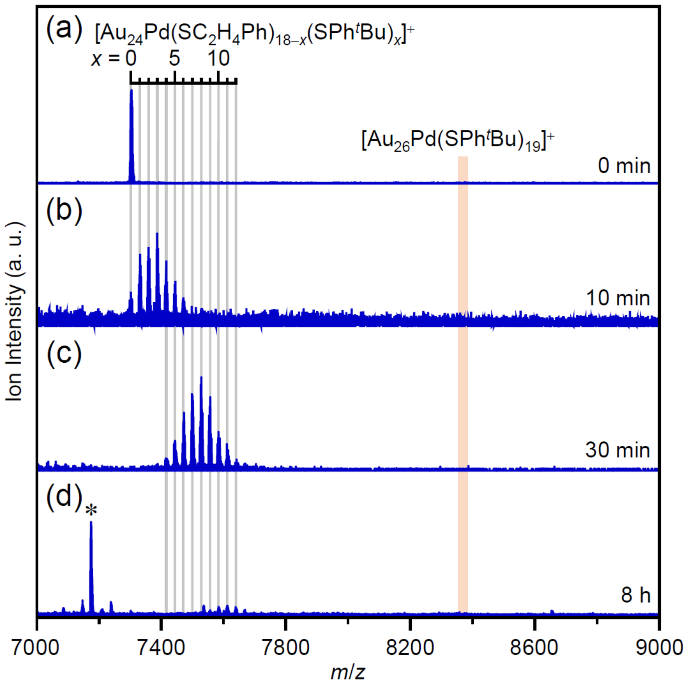

Figure S19. Positive-ion MALDI mass spectra of the products obtained by the reaction between $[Au_{24}Pd(SC_2H_4Ph)_{18}]^0$ and ^{t}BuPhSH: (a) 0 min, (b) 10 min, (c) 30 min, and (d) 8 h. A series of MALDI mass spectra indicate that the ligand-exchange reaction occurred in this experimental condition. In (d), fragment ions (*; $Au_{26}Pd(SPh^tBu)_{11}S_4$) were mainly observed, different from (a)−(c), indicating that $[Au_{26}Pd(SPh^tBu)_{20}]^0$ is more easily dissociated by laser irradiation than $[Au_{24}Pd(SC_2H_4Ph)_{18-x}(SPh^tBu)_x]^0$ NCs. Probably, $[Au_{24}Pd(SC_2H_4Ph)_{18-x}(SPh^tBu)_x]^0$ NCs are also not so stable against the laser irradiation, leading to the quite weak ion intensity of $[Au_{24}Pd(SC_2H_4Ph)_{18-x}(SPh^tBu)_x]^0$ NCs (x = 13−16) which were observed in RP-HPLC chromatogram (Figure 8(c) and S19). These results indicate that the combined use of MALDI-MS and RP-HPLC is very important to elucidate the details of the ligand-exchange mechanism while estimating the product distribution for this reaction system.

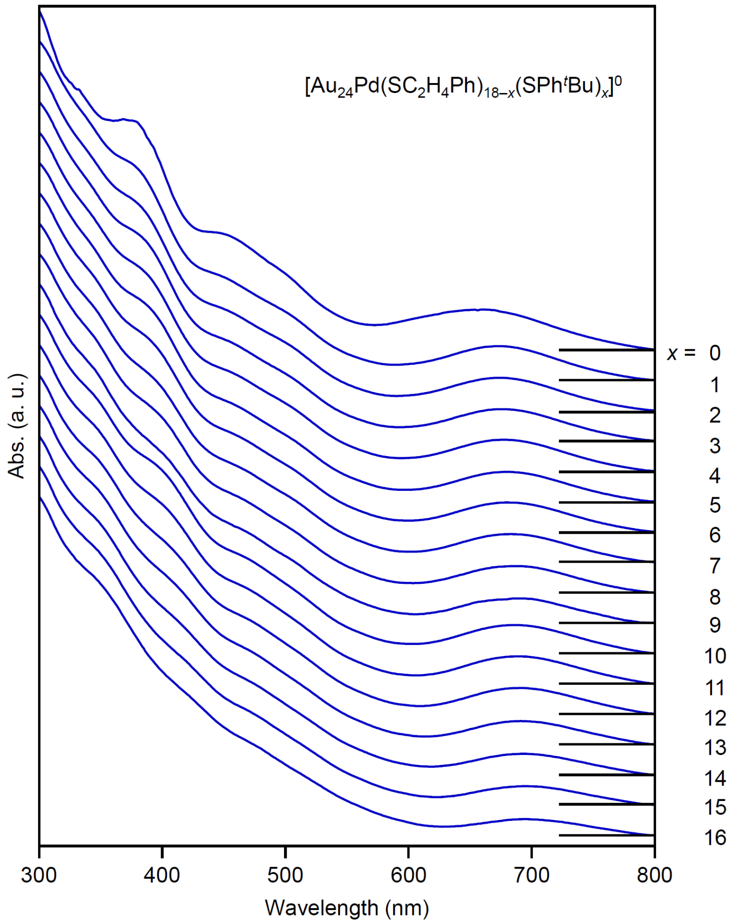

Figure S20. UV-vis optical absorption spectra of a series of $[Au_{24}Pd(SC_2H_4Ph)_{18-x}(SPh^tBu)_x]^0$ (x = 0−16) obtained by PDA detector attached to HPLC apparatus (Scheme S3(a)). Note that these optical absorption spectra are obtained not for the products with a distribution in the chemical composition, which has often been reported in the literatures,[24] but for each chemical-composition product separated by RP-HPLC (Figure 8).Only a slight change occurs with the number of the exchange ligands, which is different from the case of $[Au_{25}(SC_2H_4Ph)_{18-x}(SPh^tBu)_x]^0$ (x = 0−14; Figure 5) and $[Au_{24}Pt(SC_2H_4Ph)_{18-x}(SPh^tBu)_x]^0$ (x = 0−18; Figure 11).

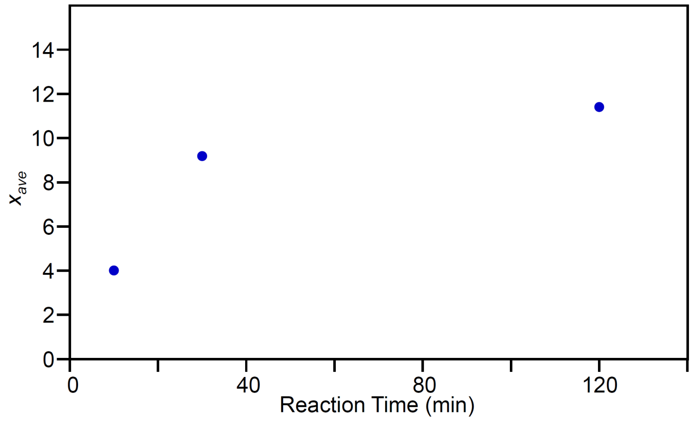

Figure S21. Plots of average number of exchanged ligands, x_{ave}, against reaction time for the reaction between $[Au_{24}Pd(SC_2H_4Ph)_{18}]^0$ and ^{t}BuPhSH (see S1.6. Analysis). The x_{ave} does not linearly increase with the reaction time, indicating that this reaction cannot be described by the pseudo-first-order reaction. Probably, the reverse reaction also occurred in parallel.

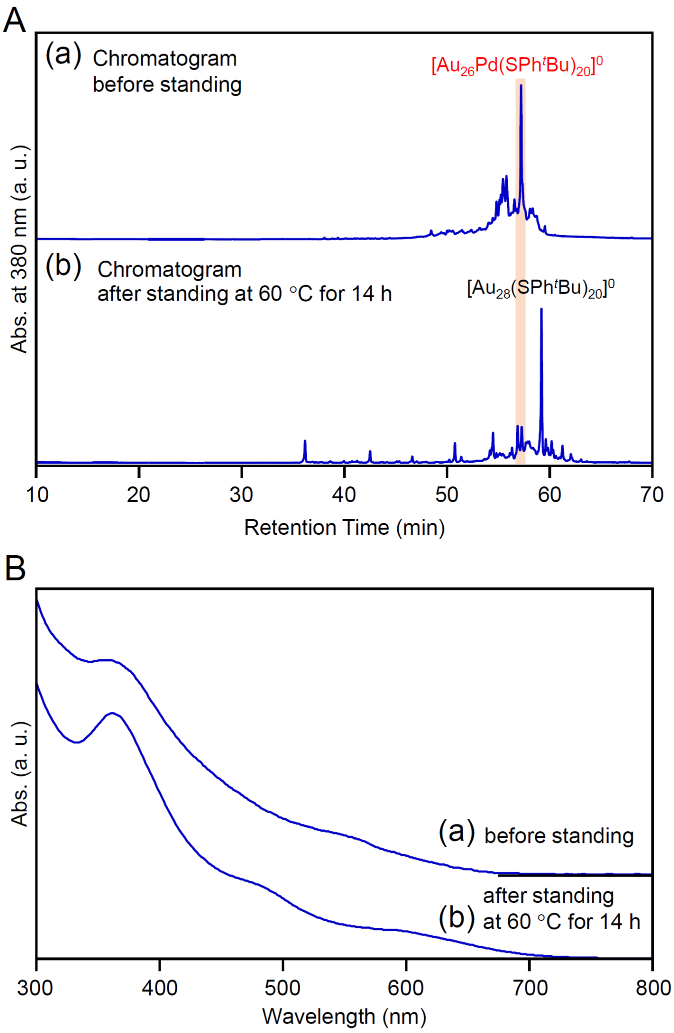

Figure S22. Stability test of $[Au_{26}Pd(SPh^tBu)_{20}]^0$ in toluene solution investigated by (A) RP-HPLC and (B) optical absorption spectroscopy; (a) before and (b) after leaving in the solution at 60 °C for 14 h. These results indicate that $[Au_{26}Pd(SPh^tBu)_{20}]^0$ cannot keep the chemical composition in toluene solution at 60 °C for 14 h. On the basis of the chromatogram (A(b)) and the optical absorption spectrum (B(b)), $[Au_{26}Pd(SPh^tBu)_{20}]^0$ changed the chemical composition into $[Au_{28}(SPh^tBu)_{20}]^0$ after standing in toluene solution at 60 °C for 14 h.

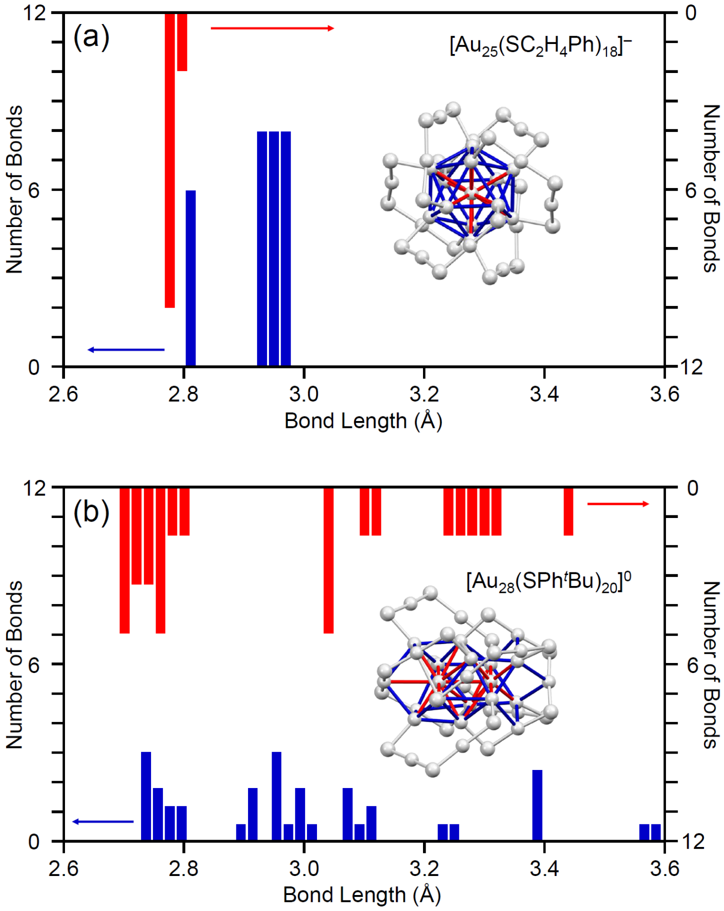

Figure S23. Bond lengths in (a) $[Au_{25}(SC_2H_4Ph)_{18}]^-$ and (b) $[Au_{28}(SPh^tBu)_{20}]^0$;[8,9] (red) central atom to surface atom and (blue) surface atom to surface atom. These results indicate that in $[Au_{25}(SC_2H_4Ph)_{18}]^-$ the bond lengths from central atom to surface atom are overall shorter than those from surface atom to surface atom, whereas in $[Au_{28}(SPh^tBu)_{20}]^0$ the bond lengths from central atom to surface atom are overall similar to those from surface atom to surface atom. These trends are caused by the difference in metal core geometry: $[Au_{25}(SC_2H_4Ph)_{18}]^-$ has an icosahedral metal core (Scheme S2(a)),[8] whereas $[Au_{28}(SPh^tBu)_{20}]^0$ has a metal core formed by a fusion of two cuboctahedrons (Scheme S2(b)).[9]

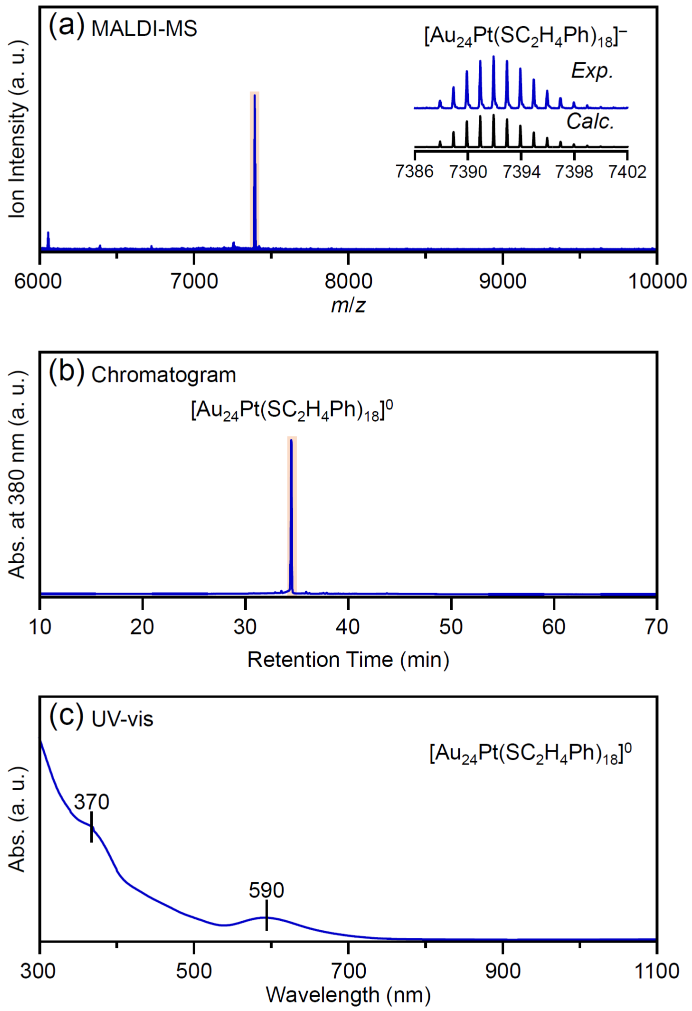

Figure S24. (a) Negative-ion MALDI mass spectrum, (b) RP-HPLC chromatogram, and (c) UV-vis optical absorption spectrum of $[Au_{24}Pt(SC_2H_4Ph)_{18}]^0$. In (a), the inset shows the comparison of the isotope pattern between the experimental and simulated spectra. In this MS, Cs^+ ion was not added to the sample solution, leading to the observation of $[Au_{24}Pt(SC_2H_4Ph)_{18}]^0$ as a $[Au_{24}Pt(SC_2H_4Ph)_{18}]^-$ due to the ionization during ESI process.[22]

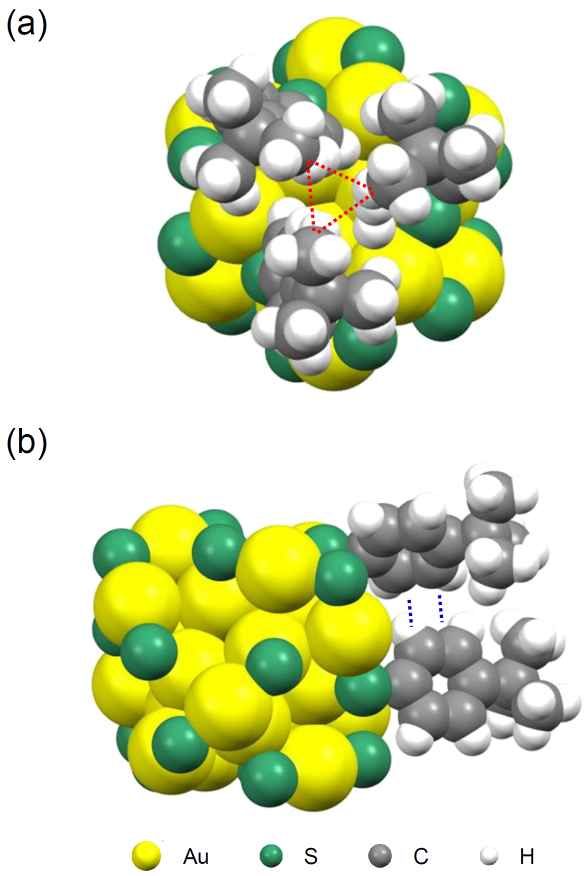

Figure S25. Space filling model drawn for a part of $[Au_{24}Pt(SPh^{t}Bu)_{18}]^{0}$; (a) top and (b) side-views. It seems that there are hydrophobic interaction (red dotted line) and C−H···π interaction (blue dotted line) between the $SPh^{t}Bu$ ligands.

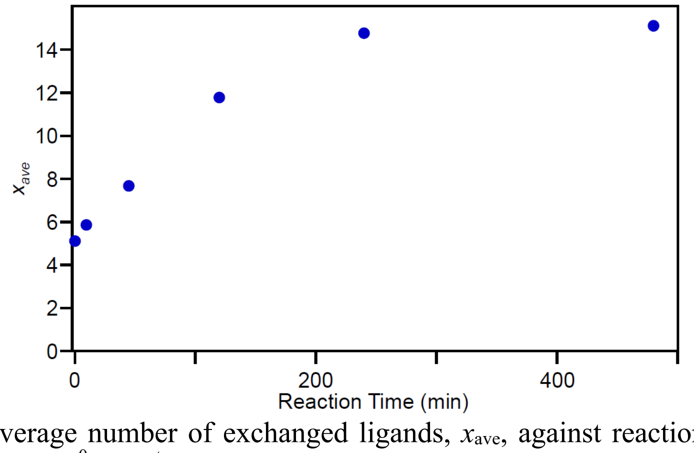

Figure S26. Plots of average number of exchanged ligands, x_{ave}, against reaction time for the reaction between $[Au_{24}Pt(SC_2H_4Ph)_{18}]^{0}$ and ^{t}BuPhSH (see S1.6. Analysis). The x_{ave} linearly increases with the reaction time of the formation of $[Au_{24}Pt(SC_2H_4Ph)_7(SPh^{t}Bu)_{11}]^{0}$ (2 h = 120 min), indicating that this reaction can be described by the pseudo-first-order reaction until 2 h. However, the x_{ave} does not linearly increase with the reaction time after that, implying that the reverse reaction also occurred in parallel after 2h.

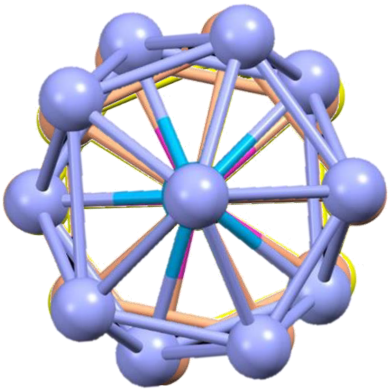

Figure S27. Comparison of the metal core structure between $[Au_{25}(SC_2H_4Ph)_{18}]^-$, $[Au_{24}Pd(SC_2H_4Ph)_{18}]^0$ and $[Au_{24}Pt(SC_2H_4Ph)_{18}]^0$.[8,12]

S5. References

1. Z. Wu, J. Suhan and R. Jin, *J. Mater. Chem.*, 2009, **19**, 622–626.
2. Y. Negishi, W. Kurashige, Y. Kobayashi, S. Yamazoe, N. Kojima, M. Seto and T. Tsukuda, *J. Phys. Chem. Lett.*, 2013, **4**, 3579–3583.
3. H. Qian, D.-e. Jiang, G. Li, C. Gayathri, A. Das, R. R. Gil and R. Jin, *J. Am. Chem. Soc.*, 2012, **134**, 16159–16162.
4. Y. Niihori, Y. Koyama, S. Watanabe, S. Hashimoto, S. Hossain, L. V. Nair, B. Kumar, W. Kurashige and Y. Negishi, *J. Phys. Chem. Lett.*, 2018, **9**, 4930–4934.
5. V. González-Ruiz, A. I. Olives and M. A. Martín, *TrAC, Trends Anal. Chem.*, 2015, **64**, 17–28.
6. Y. Negishi, S. Hashimoto, A. Ebina, K. Hamada, S. Hossain and T. Kawawaki, *Nanoscale*, 2020, **12**, 8017–8039.
7. A. Dass, A. Stevenson, G. R. Dubay, J. B. Tracy and R. W. Murray, *J. Am. Chem. Soc.*, 2008, **130**, 5940–5946.
8. M. Zhu, C. M. Aikens, F. J. Hollander, G. C. Schatz and R. Jin, *J. Am. Chem. Soc.*, 2008, **130**, 5883–5885.
9. C. Zeng, T. Li, A. Das, N. L. Rosi and R. Jin, *J. Am. Chem. Soc.*, 2013, **135**, 10011–10013.
10. C. Zeng, C. Liu, Y. Chen, N. L. Rosi and R. Jin, *J. Am. Chem. Soc.*, 2014, **136**, 11922–11925.
11. A. George, A. Sundar, A. S. Nair, M. P. Maman, B. Pathak, N. Ramanan and S. Mandal, *J. Phys. Chem. Lett.*, 2019, **10**, 4571–4576.
12. S. Tian, L. Liao, J. Yuan, C. Yao, J. Chen, J. Yang and Z. Wu, *Chem. Commun.*, 2016, **52**, 9873–9876.
13. Y. Niihori, M. Matsuzaki, C. Uchida and Y. Negishi, *Nanoscale*, 2014, **6**, 7889–7896.
14. M. Waszkielewicz, J. Olesiak-Banska, C. Comby-Zerbino, F. Bertorelle, X. Dagany, A. K. Bansal, M. T. Sajjad, I. D. W. Samuel, Z. Sanader, M. Rozycka, M. Wojtas, K. Matczyszyn, V. Bonacic-Koutecky, R. Antoine, A. Ozyhar and M. Samoc, *Nanoscale*, 2018, **10**, 11335–11341.
15. J. Peng, P. Wang, B. Wang, L. Xiong, H. Liu, Y. Pei and X. C. Zeng, *Small*, 2021, **17**, 2000627.
16. Y. Chen, C. Liu, Q. Tang, C. Zeng, T. Higaki, A. Das, D.-e. Jiang, N. L. Rosi and R. Jin, *J. Am. Chem. Soc.*, 2016, **138**, 1482–1485.
17. Y. Niihori, D. Shima, K. Yoshida, K. Hamada, L. V. Nair, S. Hossain, W. Kurashige and Y. Negishi, *Nanoscale*, 2018, **10**, 1641–1649.
18. S. Mukherjee, D. Jayakumar and S. Mandal, *J. Phys. Chem. C*, 2021, **125**, 12149–12154.
19. M. P. Maman, A. S. Nair, H. Cheraparambil, B. Pathak and S. Mandal, *J. Phys. Chem. Lett.*, 2020, **11**, 1781–1788.
20. S. Takano, S. Ito and T. Tsukuda, *J. Am. Chem. Soc.*, 2019, **141**, 15994–16002.
21. Y. Niihori, Y. Kikuchi, A. Kato, M. Matsuzaki and Y. Negishi, *ACS Nano*, 2015, **9**, 9347–9356.
22. S. Hossain, Y. Imai, D. Suzuki, W. Choi, Z. Chen, T. Suzuki, M. Yoshioka, T. Kawawaki, D. Lee and Y. Negishi, *Nanoscale*, 2019, **11**, 22089–22098.
23. D. Lin, M. Zheng and W. W. Xu, *Phys. Chem. Chem. Phys.*, 2020, **22**, 16624–16629.
24. C. Zeng, C. Liu, Y. Pei and R. Jin, *ACS Nano*, 2013, **7**, 6138–6145.
